# High-Performance Liquid Chromatography–Mass Spectrometry Analysis of Glycoalkaloids from Underexploited Solanum Species and Their Acetylcholinesterase Inhibition Activity

**DOI:** 10.3390/plants11030269

**Published:** 2022-01-20

**Authors:** Inna Popova, Belinda Sell, Syamkumar Sivasankara Pillai, Joseph Kuhl, Louise-Marie Dandurand

**Affiliations:** 1 Department of Soil & Water Systems, University of Idaho, 875 Perimeter Drive MS 2340, Moscow, ID 83844-2340, USA; sell7793@vandals.uidaho.edu; 2 Department of Entomology, Plant Pathology & Nematology, University of Idaho, 875 Perimeter Drive MS 2340, Moscow, ID 83844-2329, USA; s.sivasankarapillai@ag.tamu.edu (S.S.P.); lmd@uidaho.edu (L.-M.D.); 3 Department of Plant Sciences, University of Idaho, 875 Perimeter Drive MS 2333, Moscow, ID 83844-2340, USA; jkuhl@uidaho.edu

**Keywords:** glycoalkaloids, *Solanum caripense*, *S. melanocerasum*, *S. muricatum*, *S. nigrum*, *S. quitoense*, *S. retroflexum*, *S. sisymbriifolium*, HPLC TOF MS, acetylcholinesterase inhibition

## Abstract

*Solanum* glycoalkaloids are gaining increased scientific attention due to their bioactive potential in the defense of plants against pests and pathogens. The comprehensive glycoalkaloid profiling from the leaves, stems, and roots of seven underexploited *Solanum* species (*S. caripense*, *S. melanocerasum*, *S. muricatum*, *S. nigrum*, *S. quitoense*, *S. retroflexum*, and *S. sisymbriifolium*) was conducted using high-performance liquid chromatography–time-of-flight mass spectrometry. A total of 51 glycoalkaloids were shared among the studied *Solanum* species, with concentrations ranging from 7 to 5.63 × 10^5^ ng g^−1^. Based on the glycoalkaloid composition, plants were separated into two clusters, Cluster 1 (*S. melanocerasum*, *S. nigrum*, and *S. retroflexum*) and Cluster 2 (*S. caripense*, *S. muricatum*, *S. quitoense*, and *S. sisymbriifolium*). The inhibition activity of glycoalkaloid extracts on acetylcholinesterase showed a half-maximal inhibitory concentration (IC_50_), ranging from 0.4 (*S. nigrum* stems) to 344.9 µg mL^−1^ (*S. sisymbriifolium* leaves), that was not directly correlated to the total glycoalkaloid contents. This suggests that the composition of glycoalkaloids in the plant extract, rather than the total concentration, is a driver of biological activity. The study provides a framework for the bioprospecting of underexploited *Solanum* species for exploring bioactive glycoalkaloids and other compounds with potential pesticidal activities for the development of green bioformulation. This is the first comprehensive report on the glycoalkaloid profiles of *S. retroflexum*.

## 1. Introduction

The plants of the *Solanaceae* family contain a class of biologically active compounds called glycoalkaloids, which represent a wide class of chemical compounds composed of a C27 cholestane skeleton, to which a carbohydrate moiety of one to five monosaccharides is attached [1,2,3,4,5,6,7,8,9]. Due to their pesticidal properties, glycoalkaloids have the potential to function well in the field of plant protection. For example, *Solanum sisymbriifolium* has been demonstrated to be an efficient trap crop for the control of potato cyst nematodes, making it one of the more promising feedstocks for the production of pesticides [10,11]. Glycoalkaloids are found in more than 350 plant species, and are present in all plant organs with concentrations varying depending on the plant growth stage, environmental conditions, and specific plant species, and they can range from 18 to 10,000 mg kg^−1^ [12,13]. While a significant effort was made to identify glycoalkaloids in commercial crops, such as tomatoes and potatoes, glycoalkaloid composition is unclear in noncommercial and weedy plants, such as *S. sisymbriifolium* and *S. caripense* [14]. Still, these underexploited plants contain many glycoalkaloids that can potentially have desirable pesticidal properties. 

With the recent advance in hyphenated analytical techniques, such as high-performance liquid chromatography–time-of-flight mass spectrometry (HPLC TOF MS), the untargeted analysis of glycoalkaloids has become possible, and can facilitate the identification of novel or already-described glycoalkaloids in new plant species [15]. However, the data obtained via HPLC MS are very complex and contain a large amount of information. In combination with the lack of analytical and reference standards, this presents a challenge for identifying potential pesticidal candidates; thus, lengthy purification and isolation steps are required for activities screening. One of the approaches in analyzing the large amount of data produced through HPLC MS is the use of chemometric tools that can significantly reduce the data set and provide maximum amount of relevant chemical information, such as a subset of critical chemical compounds or a set of molecular features.

The objective of this study was to assess the applicability of a chemometrics analysis of HPLC TOF MS data for singling out chemical compounds or groups of chemical compounds with specific biological properties from underexploited *Solanum* species. HPLC TOF MS data for glycoalkaloid extracts from the roots, stems, and leaves of seven *Solanum* species (*S. caripense*, *S. melanocerasum*, *S. muricatum*, *S. nigrum*, *S. quitoense*, *S. retroflexum*, and *S. sisymbriifolium*) were analyzed, and their pesticidal potentials were assessed using the inhibition of acetylcholinesterase (AChE, EC 3.1.1.7), an enzyme that catalyzes the hydrolysis of the neurotransmitter acetylcholine [4]. 

## 2. Results and Discussions

### 2.1. Glycoalkaloids Assignment

Root, leaf, and stem extracts from seven *Solanum* species were assessed using time-of-flight MS analysis for exact molecular mass assessment, and a characteristic neutral loss pattern analysis of typical sugar moieties (pentose, deoxyhexose, and hexose) for glycoalkaloid assignment [16]. A total of 51 compounds that were assigned as glycoalkaloids were shared among all *Solanum* plants and accounted for 97–99% of the peak area in the total ion chromatograms (Table 1). Chromatographic retention times for most of the glycoalkaloids clustered at around 22–25 min, suggesting that they had closely related chemical structures and polarities (Figure S1). The majority of the glycoalkaloids were baseline separated by the HPLC elution program; however, several coeluting or overlapping peaks were also present. In this case, mass spectra deconvolution was used to distinguish the mass spectra belonging to different compounds using the Agilent Profiler software package. For example, the spectra of two co-eluting glycoalkaloids at 25.67 min were resolved to obtain the individual fragmentation pattern of compounds **22** (*m*/*z* 1105.54) and **23** (*m*/*z* 1046.54).

The molecular weight of the pseudo-molecular ions for the assigned glycoalkaloids ranged from 884.50 to 1563.75 *m*/*z*, with the majority of the compounds being in the 1000–1100 *m*/*z* range. Among the 51 compounds, eight isomeric pairs were identified. Similarities in fragmentation patterns and the relative abundances of fragments for the majority of isomeric pairs suggested that these are stereo rather than structural isomers. The glycoalkaloids **6** and **17** with the molecular ion [M + H]^+^ 868.50 detected at 22.19 and 24.67 min, respectively, is one such example. Similarly, compounds **1** and **15** with the molecular ion [M + H]^+^ 884.5042 eluting at 17.05 and 26.35 min, respectively, with the same fragmentation pattern and fragment abundance, are likely stereoisomers. The presence of multiple isomeric pairs is consistent with the previous findings for glycoalkaloids in plants where multiple isomers are usually reported for the same molecular weight compounds [33]. 

**Figure 1:**
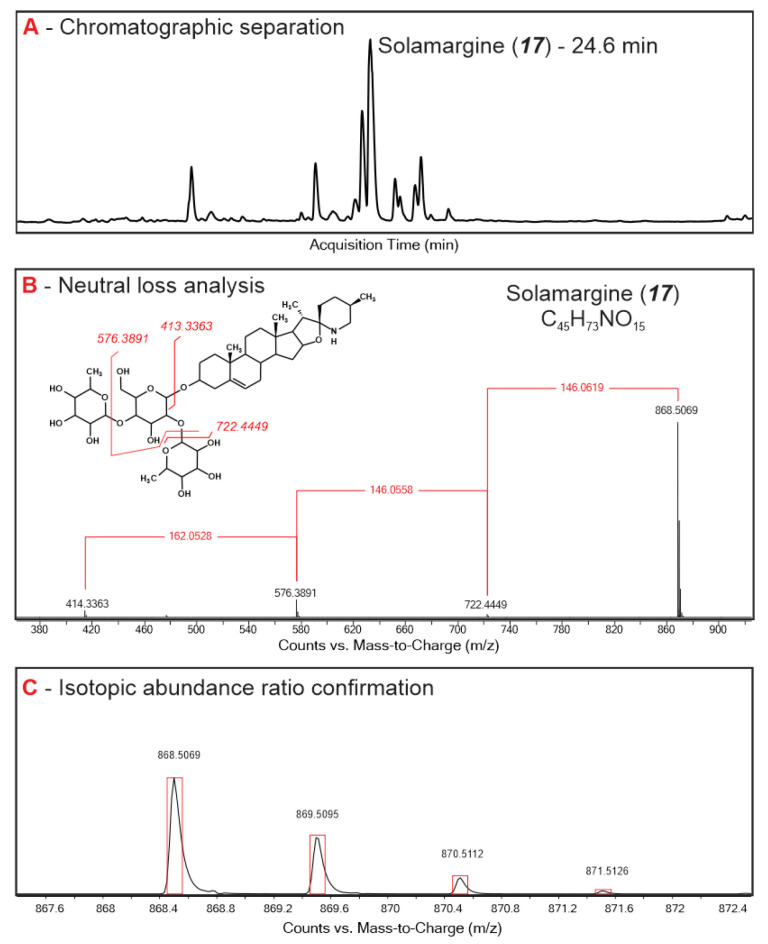
Typical HPLC TOF MS chromatogram of *S. sisymbriifolium* (**A**), fragmentation pattern and sugar moiety assignment (**B**), and stable isotope ratio analysis (**C**) of assigned glycoalkaloids.

For the majority of the assigned glycoalkaloids, steroidal aglycone was identified based on the neutral sugar loss and the exact mass of the corresponding fragment present in the MS spectra (Figure 1, Table 1). A pseudo-molecular ion of 414.33, corresponding to solanidine aglycone, was present in the MS spectra of 13 assigned glycoalkaloids. Solanidine, also known as solatubin or solanid-5-en-3-ol, contains an oxazaspirodecane ring system and is present in solamargine- and solasonine-type glycoalkaloids [19,34]. An aglycone with a molecular mass of 430.33 was present in four assigned glycoalkaloids. This aglycone likely corresponds to the hydroxylated solanidine [19]. A fragment corresponding to jurubidine, a cyclized 3β-aminospirostane aglycone (*m*/*z* of 416.35) was present in three compounds. For the remaining assigned glycoalkaloids, the fragmentation pattern in the lower mass ranges was not strong enough to accurately identify the aglycone. Based on the neutral loss analysis (Table 1), 20 and 10 assigned glycoalkaloids were determined to contain a triose and teraose sugar moiety, respectively [16,35]. While the specific sugars and their linkage to the aglycone molecule cannot be accurately assigned, based on the consequent neutral loss mass, mono-, di-, tri-, and tetrameric sugar forms that could be obtained by combining pentose, hexose, and deoxyhexose were suggested (Table 1) [35].

The assigned glycoalkaloids were tentatively identified based on reference standards, the comparison of MS spectra with those reported in the literature, accurate mass data, and the chemical abstracts service database for the occurrence of the specific compounds [36]. Compounds **15** and **17** were identified against the analytical standards for solamarine and solamargine, respectively. Compounds **1** and **29** were identified as isomers of hydroxy-solamargine based on the molecular ion [M + H]^+^ of 884.50 and the aglycone fragment of 430.33, which corresponds to hydroxylated solasodine [19]. Hydroxy-solamargine was previously reported in the fruits of *S. nigrum*, as well as in *S. melongena*, in correlation with solamargine [17,18]. Compound **2** has been described in *S. melongena* previously as malonyl-solanandaine [20]. The high-resolution mass spectra of compound **2** suggests the presence of an aglycone fragment with an *m*/*z* of 430.33 that corresponds to solasodine. Compound **3** can be assigned as arudonine, which was previously detected in *S. melongena* and *S. arundo* [20,21,22]. Compound **6** is structurally similar to solamargine and is likely its stereoisomer. Compound **7**, solaviaside B (C_26_H_43_O_11_), has been described previously in *S. nigrum*, as well as *S. viarum* [23,24]. Compound **9** is structurally similar to indioside D, which has been previously described in *S. indicum* and *S. nigrum* [25,26]. Compounds **12** and **13** have been reported in *S. nigrum* and can be assigned as isomers of solanigroside H [27]. Compound **16** and **21** has been reported in *S. sycophanta*, and were identified as sycophantine and hydroxy-sycophantine, respectively [21]. Compound **20**, solanigroside Y5, was previously reported in *S. nigrum* [24]. Based on the previously reported composition of *S. nigrum* and the MS spectra of the detected compounds, compounds **26** and **28** were assigned as malonyl-solamargine and solanigroside E, respectively [20,28]. Compound **31** was described previously in the ripe fruits of *S. lycocarpum*; however, the structure was not assigned [37]. Compound **35** was described in *S. nigrum*, *S. myriacanthum*, and *S. anguivi* as anguivioside XI [29,30,31]. A compound with the same molecular mass as compound **36** was detected in *S. nigrum* and *S. muricatum*; however, no mass spectra were provided [38]. Compound **40** can be assigned as lyconoside II based on the glycoalkaloid composition of the *S. lycocarpum* fruit [32]. Compound **43** is structurally similar, based on the mass spectra, to solanigroside D, which has been detected in *S. nigrum* previously [27]. 

**Figure 2:**
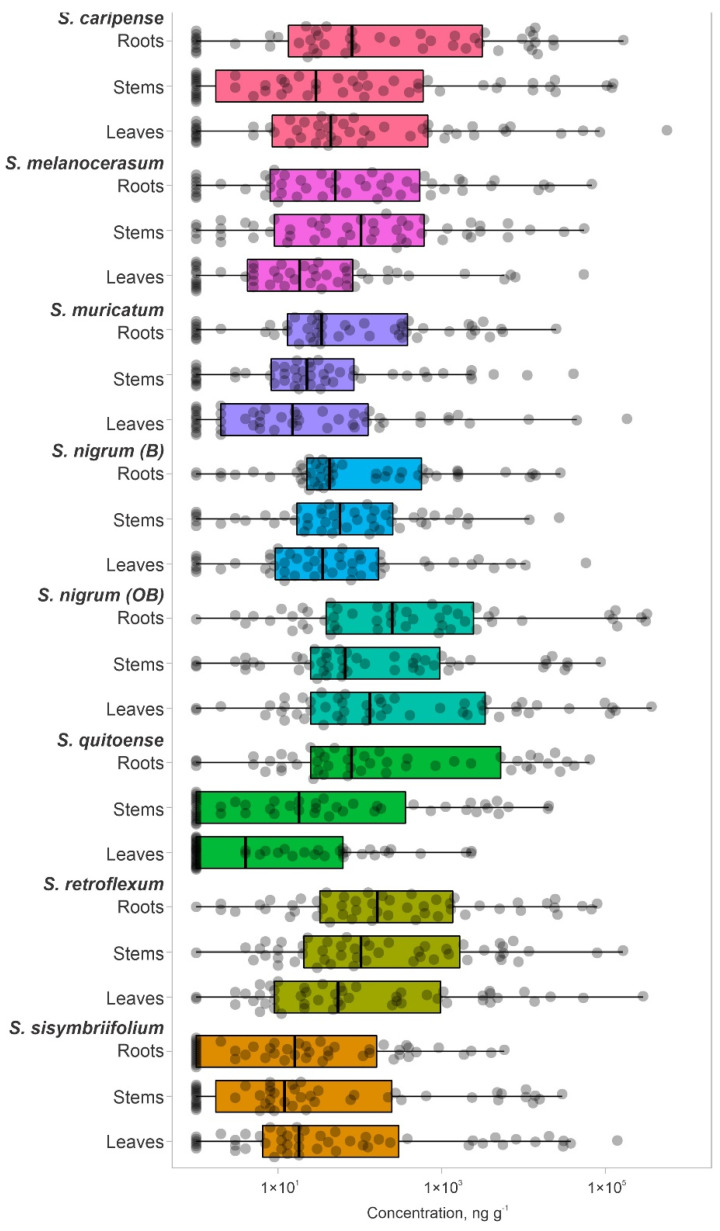
Distribution and relative amounts of individual glycoalkaloids (Table 1 and Table S1) in shoots and roots of *Solanum* plant extracts.

Since the exact glycoalkaloid composition of the analyzed plants is unknown, the total number of glycoalkaloids in the plant extracts could be higher. However, this subset of 51 glycoalkaloids is representative of the major glycoalkaloid constituents in the set of analyzed plants. Extracts of tested *Solanum* species were dominated by a small subset of glycoalkaloids (Figure 2, Table S1). Extracts from both shoots and roots contained glycoalkaloids with a similar detection frequency (Figure 2, Table S1). The cumulative amounts of glycoalkaloids in the shoots were significantly higher than in the roots, as reflected by total the peak areas for *S. caripense*, *S. melanocerasum*, *S. muricatum*, *S. nigrum*, *S. retroflexum*, and *S. sisymbriifolium*. For example, the amounts of glycoalkaloids in the shoots of *S. sisymbriifolium* and *S. muricatum* were 24.6- and 12-fold higher than in the roots, respectively. These data are consistent with the previously reported finding that glycoalkaloid concentrations are the highest in leaves and fruits. *Solanum quitoense* was the only species that had 30% higher total glycoalkaloid amounts present in the roots compared to the shoots. This was likely due to the higher amounts of four major glycoalkaloid compounds (**14**, **23**, **31**, and **32**) that were detected in roots. 

The major glycoalkaloid compounds detected in *S. nigrum* were **8**, **15**, **17**, **19**, **20**, **26**, **28**, **46**, and **47** (Table 1 and Table S1). Compounds **20**, **28,** and **46** were present in all parts of the plant at relatively high amounts. Shoots contained compounds **19** and **20** predominantly, while roots were dominated by compound **47**. *Solanum melanocerasum*’s main glycoalkaloids were **20**, **24**, **25**, **28**, **32**, **36**, **38**, **42**, and **46**, with compound **20** present in all plant parts. The roots contained only four major compounds, **20**, **25**, **38**, and **46**, while the shoots contained three major compounds, **20**, **24**, and **28**. The major glycoalkaloids detected in *S. retroflexum* were **4**, **15**, **17**, **20**, **28**, **38**, and **46**. Consistent glycoalkaloid diversity was observed throughout the plant, with most of the predominant glycoalkaloids present in all plant parts. Roots had compounds **15**, **17**, **20**, **28**, **38**, and **46**, while shoots contained three predominant compounds, **15**, **17**, **20**, **28**, and **46**.

The main glycoalkaloids detected in *S. caripense* were **5**, **8**, **11**, **12**, **14**–**17**, and **26**. Compounds **5**, **14**, **17**, and **26** were predominant in leaves. The roots contained three major compounds, **12**, **16**, and **26**. The major glycoalkaloids detected in *S. muricatum* were **3**, **8**, **11**–**13**, **15**–**18**, **26**, **32**, **37**, and **39**. The roots had the highest glycoalkaloid diversity with compounds **8**, **11**–**13**, **15**–**18**, **26**, **32**, and **37** present in relatively large amounts. The shoots had only four major glycoalkaloids, **15**–**17** and **26**. Both solamargine and solasonine have previously been reported in *S. muricatum* [39]. The glycoalkaloid composition of *S. quetoense* was uniform across the detected compounds, with multiple assigned glycoalkaloids (**4**, **14**, **16**, **17**, **23**, **26**, **31**, **32**, **40**, **41**, **45**, and **48**) being in the same level. Unlike other plant species, the roots had the highest diversity of glycoalkaloids, with compounds **14**, **16**, **17**, **23**, **32**, **41**, **45**, and **48** being present in relatively large amounts. The major glycoalkaloids detected in *S. sisymbriifolium* were **1**, **2**, **6**, **16**, **17**, **21**, **26**, **29**, **32**, **42**, **49**, and **50**. The roots had four major compounds, **16**, **32**, **49**, and **50**. The shoots had compounds **1**, **2**, **16**, **17**, **26**, and **42**. 

The glycoalkaloid concentrations, quantified based on the solamargine standard, were in the range of 7–5.63×10^5^ ng g^−1^ (DW basis) (Figure 2). For most of the plants, the median glycoalkaloid concentration was similar for shoots and roots; however, the distribution of specific glycoalkaloid concentrations differed (Figure 2). For example, the *S. sisymbriifolium* median for glycoalkaloid concentrations was higher for shoots than for roots. The glycoalkaloid concentration in roots clustered close to the median, while in shoots, an additional cluster in the third quartile was observed. This is consistent with the lower total glycoalkaloid content for *S. sisymbriifolium* roots. Most of the *S. caripense*, *S. quitoense*, and *S. sisymbriifolium* glycoalkaloids concentrations were close to the median and third quartiles (Figure 2). The higher concentrations accounted for 97–98% of the total glycoalkaloid content, represented by 7–17 individual glycoalkaloids. The median for *S. quitoense* shoots was lower than for roots due to a large number of glycoalkaloids with low concentrations resulting in a longer plot. This is also reflected in the skewed data and relatively low first-quartile value. For the *S. quitoense* roots, an opposite trend was observed, with the majority of the glycoalkaloids having concentrations above the median value and relatively evenly distributed third and fourth quartiles.

### 2.2. Glycoalkaloids Clustering in Solanum Plants

**Figure 3:**
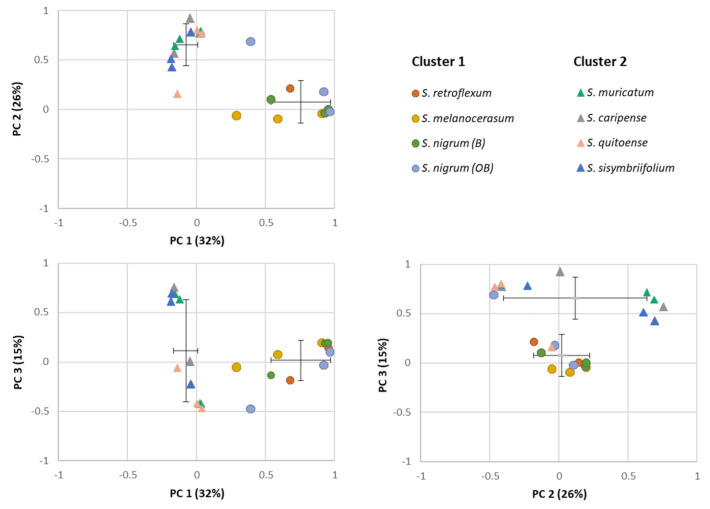
Principal component analysis (PCA) plots for individual glycoalkaloids in the shoots and roots of *Solanum* plant extracts.

The similarities among the *Solanum* plants’ shoot and root extracts were assessed via PCA based on the composition and abundance of individual glycoalkaloids (Figure 3). Total variance (73%) was explained by first three principal components, with PC1, PC2, and PC3 accounting for 32, 26, and 15%, respectively. Based on the PCA loading plot, a separation of extracts on two clusters was observed: Cluster 1 included *S. melanocerasum*, *S. nigrum*, and *S. retroflexum*, and Cluster 2 included *S. caripense*, *S. muricatum*, *S. quitoense*, and *S. sisymbriifolium*. For the Cluster 1 plants (*S. melanocerasum*, *S. nigrum*, and *S. retroflexum*), the primary glycoalkaloids present in all plants were **15**, **17**, **20**, **28**, and **46**. In the Cluster 2 plants, three predominant compounds (**16**, **17**, and **26**) that belong to solanidine-type glycoalkaloids were present across all the species [19,34]. The clustering is consistent with the fact that *S. nigrum*, *S. retroflexum*, and *S. melanocerasum* belong to the Morelloid clade and have been shown to be genetically related based on morphological and molecular data [40,41]. Cluster 2 plants do not belong to the same clade. However, *S. muricatum* has been shown to be related to wild-species *S. caripense*, and thus can share a similar glycoalkaloid composition [42]. The basis of its relationship to other Cluster 2 plants is not clear, as *S. quitoense* belongs to the Lasiocarpa clade and *S. sisymbriifolium* appears to be closely related to the Androceras/Crinitum clade [43,44]. While the clustering of *Solanum* plants based on the composition of their secondary metabolites profiles has been previously used for the chemotaxonomy of *Solanum* plants, to the best of our knowledge, the use of glycoalkaloids for *Solanum* biological activity clustering has not been investigated yet for the selected *Solanum* species [45].

**Figure 4:**
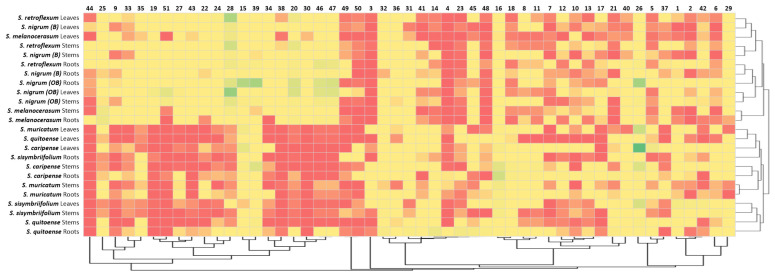
Heat map analysis of assigned glycoalkaloids (Table 1) in the extracts of *Solanum Spp,* roots, stems, and leaves.

To confirm the observed clustering of *Solanum* plants based on their glycoalkaloid content, the Pearson’s correlations of the glycoalkaloid distributions in the shoots and roots were analyzed (Table 2). Strong correlation coefficients within the two clusters signify the applicability of glycoalkaloids as a chemical class for relating the species to the *Solanum* family. Specifically, the correlation coefficients for the Cluster 1 plants (*S. melanocerasum*, *S. nigrum*, and *S. retroflexum*) leaf glycoalkaloids ranged from 0.853 *** to 0.994 ***. For the Cluster 2 plants (*S. caripense*, *S. muricatum*, *S. quitoense*, and *S. sisymbriifolium*), the association amongst the glycoalkaloids in leaves was also strong, with coefficients ranging from 0.534 *** to 0.992 ***. Similarly, the association among root glycoalkaloids in the studied *Solanum* plants was present, though weaker, for the two identified clusters, with correlation coefficients ranging from 0.124 to 0.524 *** for Cluster 1 and from 0.037 to 0.966 *** for Cluster 2. A heat map analysis of the glycoalkaloids contributing to the *Solanum* species corroborate the presence of two distinct clusters (Figure 4).

The clustering of plants based on their glycoalkaloid composition was confirmed using a Czekanowski similarity analysis (Table 3). Several strong correlations were noted, as reflected by the correlation indices close to 1. Generally, *S. nigrum*, *S. melanocerasum*, and *S. retroflexum* were more closely correlated, with indices averaging at 0.33. In fact, the glycoalkaloid composition of *S. melanocerasum* leaves was very close to *S. nigrum (B)* leaves in terms of the amount and distribution of glycoalkaloids (0.83). *S. nigrum (OB)* leaves were very similar to *S. retroflexum* leaves (0.68). Plant extracts from *S. caripense*, *S. muricatum*, *S. quitoense*, and *S. sisymbriifolium* were similar based on their glycoalkaloid composition. However, the similarity indices were lower than for the first set of plants, with the highest being 0.63.

### 2.3. Glycoalkaloids Acetylcholinesterase Inhibition Activity

**Figure 5:**
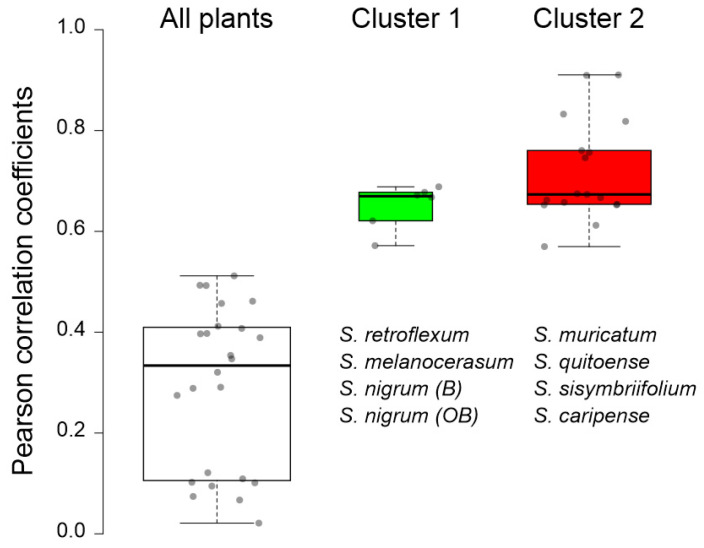
Correlation (expressed as Pearson correlation coefficients) between the half-maximal inhibitory concentration (IC_50_) of *Solanum* plant extracts and the concentrations of selected glycoalkaloids (Table 1). The IC_50_ values for Cluster 1 plants were correlated with the concentrations of compounds **9**, **10**, **12**, **13**, **17**, **35**, and **51**; the IC50 values for Cluster 2 plants were correlated with the concentrations of compounds **1**, **2**, **6**, **14**, **15**, **20**, **21**, **23**, **26**, **29**, **30**, **34**, **38**, **39**, **41**, **43**, **46**, and **47** (Table 1).

To evaluate the correlation between plant clustering, based on the glycoalkaloid content and composition, and the pesticidal potential, plant methanolic extracts were tested for the inhibition of AChE (Table 4). The inhibition of AChE results in the disruption of neurotransmission, and, in the case of nematodes, the cessation of nerve impulses and, ultimately, death [4,46]. Inhibition, as reflected by IC_50_ values, ranged from 39 to 93% when extracts were used at the nominal concentration of glycoalkaloids (as calculated on a solamargine basis). The corresponding IC_50_, calculated on the total glycoalkaloid basis, varied from 0.4 to 344.9 µg total glycoalkaloid mL^−1^. These values are in the same order of magnitude as for the previously reported IC_50_, where crude extracts of eleven *Solanums* spp. were tested [20,47]. Generally, the extracts of leaves exhibited the highest inhibition potential, while extracts from roots had the lowest inhibition of AChE. This trend can partially be explained by a concentration-dependent effect because, for most of the tested species, leaves contained the highest concentrations of glycoalkaloids. However, the correlation between the total glycoalkaloid concentration in the plant tissue extracts and half-maximal inhibitory concentration (IC_50_) values was not strong (r = 0.1520), and as a result, the total glycoalkaloid concentration cannot be used as a predictor of the pesticidal potential (Figure 5). Separating plants into two groups (Group 1 and Group 2) based on the similarity of their glycoalkaloid composition resulted in significantly better correlation and pesticidal potential prediction (Figure 5). Particularly, the strong correlation between seven major glycoalkaloids and the IC_50_ values was observed for *S. caripense*, *S. muricatum*, *S. quitoense*, and *S. sisymbriifolium* (Cluster 2). The concentration of 19 other GAs also exhibited a strong correlation with the *S. melanocerasum*, *S. nigrum (B)*, *S. nigrum (OB)*, and *S. retroflexum* (Cluster 1) IC_50_ values. These results indicate that not all glycoalkaloids contribute equally to the overall pesticidal potential. In fact, the strong correlations between the IC_50_ values and glycoalkaloid concentrations were not observed for the most abundant glycoalkaloid. This suggests that structure, rather than the total amount of glycoalkaloids, plays the primary role in determining the pesticidal potential. These results are consistent with previously reported findings that both aglycone type and the sugar moiety of glycoalkaloids have a significant effect on AChE inhibition [48]. For example, α-solamargine has a chacotriose sugar moiety and α-solasonine has a solatriose sugar moiety, and the inhibitory activity of α-solasonine was at least 10 times lower [48].

## 3. Materials and Methods

### 3.1. Solanum *spp*.

Seeds for *S. caripense* (Tzimbalo Melon Pear), *S. muricatum* (Pepino Melon), *S. melanocerasum* (*S. scabrum*, Garden Huckleberry), *S. nigrum* (Blackberry), *S. quitoense* (Naranjilla), *S. retroflexum (S. burbankii*, Wonderberry), and *S. nigrum* (Otricoli Orange Berry) were obtained from Baker Creek Heirloom Seeds (Mansfield, MO, USA). Plants were germinated on Petri dishes, transplanted to potting soil, and kept under greenhouse conditions 18 ± 2 °C (daytime), 14 ± 2 °C (nighttime), with a 16:8 h light–dark period. *S. sisymbriifolium* plants were obtained from Dr. Joseph Kuhl as 6-week-old plants. At 6 weeks after transplanting, plants were harvested by gently separating them from the soil. After washing in DI water, plants were sectioned into roots, leaves, stems, and flowers, and buds when these were present. The separated plant parts were pat dried, frozen in liquid nitrogen, and freeze dried. Dry tissues were pulverized using a cyclone mill (UDY Corporation, Fort Collins, CO, USA) and kept at −20 °C until extraction.

### 3.2. Extraction of Glycoalkaloids

Plants tissues (0.2 g) were homogenized with 10 mL of 90% methanol in Omni Prep homogenizer (Omni Int, Kennesaw GA, USA) at 10 K rpm for 10 min. The suspension was centrifuged at 4000× *g* rpm and 18 °C for 10 min. The extraction was repeated, and all extracts were combined. Chlorophyll was removed via liquid–liquid extraction with hexane (10 mL) using an end-to-end shaker (10 min). The hexane fraction containing chlorophyll was removed by aspirating the top layer after centrifuging the solution at 2400× *g* rpm at 18 °C for 5 min. The hexane extraction was repeated. The methanolic fraction of the extract was then diluted with DI water (600 mL) to obtain less than 3% methanol content. The diluted extract was subjected to solid-phase extraction on an Oasis HLB (3 mL, 60 mg, 30 µm) (Waters Corp., Milford, MA, USA). The cartridges were preconditioned with 3 mL of methanol and equilibrated with 3 mL of water. The samples were loaded into the column at 25 mm Hg negative pressure, and cartridges were washed with water and eluted with 3 mL 85% methanol using gravity.

### 3.3. HPLC TOF MS Analysis of Glycoalkaloids

The analysis of the GAs was performed using an Agilent 1200 Series HPLC coupled to an Agilent G6230 ESI TOF MS (Agilent, Santa Clara, CA, USA). The chromatographic separation of glycoalkaloids was performed on an Extend-C18 3.5 µm, 2.1 mm × 100 mm (Agilent Technologies Inc., Santa Clara, CA, USA) reversed-phase chromatographic column. The mobile phase consisted of 0.1% formic acid in water (solvent A) and 0.1% formic acid in acetonitrile (solvent B). The gradient program started with isocratic elution using 5% B for 2 min, followed by a linear gradient to 35% B from 2 to 35 min, followed by a linear gradient to 98% over 10 min, kept at 98% for 5 min, and re-equilibrated back to the initial mobile phase composition over 10 min. The column was maintained at 30 °C. The injection volume was 5 μL. The flow rate was 0.2 mL min^−1^.

Electrospray ionization was operated in positive mode. The absolute value for electrospray ionization potential was 3500 V. The collision-induced dissociation potential was set at 150 and 250 V to analyze the spectra for molecular ion and fragmentation patterns, respectively. The gas temperature was 350 °C, the drying gas (N_2_) flow rate was 10 L min^−1^, and the nebulizer pressure was 2.4 × 10^5^ Pa. The analyses were conducted in a centroid mode within an *m*/*z* range from 100 to 1700 amu. The quantification of the total glycoalkaloid concentration was performed based on the external calibration curve constructed for solamargine based on the pseudo-molecular ion [M + H]^+^.

HPLC TOF MS chromatograms were processed in MassHunter B.08.00 Agilent Profinder software (Agilent Technologies Inc, Santa Clara, CA, USA). The assignment of glycoalkaloids was performed based on neutral loss analysis using fragmentation and isotopic patterns for each compound [16,35]. Neutral loss corresponding to mono-, di-, tri-, and tetrameric sugar moieties that could be obtained by combining pentose, hexose, and deoxyhexose units were considered to be with the error of less than 3 ppm.

### 3.4. Acetylcholinesterase Assay

The measurement of acetylcholinesterase (EC number 3.1.1.7) activity was conducted in a 96-well microplate format according to a method previously described, with some modifications [49]. Different volumes (5–1000 µL) of plant extracts in 85% methanol samples were added to microplate wells, evaporated to dryness, and reconstituted in 200 µL of sodium phosphate buffer (100 mM, pH 7.4). Then, 50 µL of Ellman’s reagent (1 mM acetylthiocholine and 0.3 mM 5,5′-dithiobis (2-nitrobenzoic acid) in (100 mM pH 7.4 phosphate buffer) was added to each well. Prior to start of the analysis, 50 µL of acetylcholinesterase from *Electrophorus electricus* (1 unit mL^−1^) was added to each well. The absorbance at 405 nm was measured every minute for 15 min using SpectraMax M Series Multi-Mode Microplate Readers (Molecular Devices, LLC., San Jose, CA, USA). The rate of the reaction was calculated as a slope of the kinetics curve over the linear range. A negative control was run with each batch, substituting the plant extract for 85% methanol and treating it in a similar manner. The inhibition of the reaction by the plant extracts was quantified in terms of half-maximal inhibitory concentration (IC_50_), i.e., the concentration at which a substance exerts half of its maximal inhibitory effect. The IC_50_ was calculated using a four-parameter regression model: *y* = *d* + (*a* − *d*)/(1 + (*x*/IC_50_)*^b^*), where *y* is the response and *x* is the concentration, *a* and *d* are lower and upper asymptotes, respectively, and *b* is the steepness of the linear portion of the curve [50].

### 3.5. Data Analysis

All extractions and enzyme assays were performed with at least three replicates. The heat map analysis was conducted using a ClustVis web tool for visualizing the clustering of multivariate data that is based on the heatmap R package (version 0.7.7) [51]. The bean plots were generated using a BoxPlotR web tool for the generation of box plots [52]. Czekanowski similarity indices (*s_c_*) were calculated as the ratio of the double sum of the lesser scores of each glycoalkaloid to the sum of all glycoalkaloids in the specific sample and were used as a measure of similarity between two categorical distributions [53]. The correlation between specific glycoalkaloids and inhibition activities were calculated using Pearson’s correlation in JASP, an open-source graphical software package for basic statistical procedures [54].

## 4. Conclusions

The presented study provides a framework for creating data sets for further biologically active compound identification and analysis. Specifically, using this framework we were able to demonstrate that the composition of glycoalkaloids in plant extracts rather than the total concentration is a driver of inhibitory activity. The *S. sisymbriifolium* leaf extract with the highest AChE inhibition had a unique glycoalkaloid, tentatively identified as malonyl solanandaine, which was not identified in the other *Solanum* species used in this study. This chemometric study will help in the bioprospecting of underexploited *Solanum* species for exploring bioactive compounds with potential biological activities for the development of green bioformulation. This, as in the case of this study, can be a useful tool for screening for the potential pesticidal activities of plant tissues. Recently, the multivariant analysis was identified as a critical tool in the chemotaxonomy of eggplants (*S. melongena*) and their wild relatives [55]. However, this is, to the best of our knowledge, the first report of the analysis of glycoalkaloids across the seven studied *Solanum* species. In addition, the proposed workflow can be applied to other areas of study, such as chemophenetic analysis, which is an emerging concept relating to plant chemosystematics, and chemotaxonomy, which is based on the plant specialized metabolites profile [56].

## Figures and Tables

**Table 1:** Major glycoalkaloids identified in all *Solanum* plant extracts. Sugar moieties are proposed based on the neutral loss analysis of deconvoluted mass spectra. Pentose—Pen; deoxyhexose—DeoxyH; hexose—Hex; *S. nigrum* (OB)—SN(OB); *S. nigrum* (B)—SN(B); *S. melanocerasum*—SMe; *S. retroflexum*—SR; *S. sisymbriifolium*—SS; *S. quitoense*—SQ; *S. caripense*—SC; *S. muricatum*—SMu; roots—R; leaves—L; stems—S.

Compound * r.t		[M + H]^+^	Major Fragments	Sugars Neutral Loss	Detected in Plant Extract (>5% of TIC)	Glycoalkaloid Assignment
**1**	17.05	884.50	738.43, 592.38, 453.74, 430.32	-DeoxyH, -DeoxyH, -Hex	SS S, SS L	hydroxy-solamargine [17,18,19]
**2**	18.64	970.49	824.44, 738.44, 592.38, 496.74, 430.33	-DeoxyH, -DeoxyH, -Hex	SS L	malonyl-solanandaine [20]
**3**	19.50	1000.54	722.45, 576.38, 511.76, 414.33	-Pen + DeoxyH, -DeoxyH, -Hex	SMu R, SMu S	arudonine [20,21,22]
**4**	20.88	1045.52	899.46, 753.40, 591.35	-DeoxyH, -DeoxyH, -Hex	SQ R	
**5**	22.02	888.55	742.48, 596.41, 434.35	-DeoxyH, -DeoxyH, -Hex	SC L	
**6**	22.19	868.50	722.44, 576.39, 414.33	-DeoxyH, -DeoxyH, -Hex	SS S	solamargine isomer
**7**	22.53	1063.53	901.74, 755.42, 593.37	-Hex, -Hex	SMu R	solaviaside B [23,24]
**8**	22.65	1046.55	722.45, 576.40, 534.78, 414.34	-2Hex, -DeoxyH, -Hex	SMu R	
**9**	22.80	1065.54	903.49, 757.43, 595.38, 357.11	-DeoxyH, -Hex, -Hex	SMe S, SMe L	indioside D [25,26]
**10**	22.96	903.56	357.11, 275.10		SMe L, SC R	
**11**	23.02	1046.57	722.45, 576.40, 534.79, 414.35	-2Hex, -DeoxyH, -Hex	SC S, SMu R	
**12**	23.32	1048.57	724.46, 578.41, 535.78, 416.35	-2Hex, -DeoxyH, -Hex	SC R, SMu R	solanigroside H [27]
**13**	23.74	1048.57	724.46, 578.41, 535.78, 416.35	-2Hex, -DeoxyH, -Hex	SC R	solanigroside H isomer [27]
**14**	23.80	1030.54	884.47, 738.42, 576.37, 526.76, 414.32	-DeoxyH, -DeoxyH, -Hex, -Hex	SQ R, SQ S, SQ L	
**15**	23.96	884.50	722.45, 576.39, 414.34	-DeoxyH, -Hex, -Hex	SN(OB) R, SN(OB) S, SN(B) R, SR R, SQ S, SQ L, SC S, SC L, SMu R, Smu L	solamarine
**16**	24.22	1000.54	722.44, 576.38, 511.76, 414.32	-Pen, -DeoxyH, -DeoxyH, -Hex	SS R, SS S, SS L, SC R, SC S, SMu R, Smu S, Smu L	sycophantine [21]
**17**	24.67	868.5067	722.4465, 576.3889, 414.3360	-DeoxyH, -DeoxyH, -Hex		solamargine
**18**	24.90	1002.57	886.52, 724.46, 578.41, 416.35		SS S, SC R, SC S, SMu R	
**19**	25.34	1373.63	1211.57, 741.45, 579.39, 413.14	-Hex, -2Hex + Rha, -Hex	SN(B) S, SN(B) L	
**20**	25.51	1227.60	1065.55, 741.45, 579.39	-Hex, -Hex, -Hex	SN(OB) R, SN(OB) S, SN(OB) L, SN(B) R, SN(B) S, SN(B) L, SMe R, SMe S, Sme L, SR R, SR S, SR L	solanigroside Y5 [24]
**21**	25.52	1016.54	738.4398, 592.3824, 525.3006, 430.3296	-Pen + DeoxyH, -DeoxyH	SS R, SS S, SQ R, SQ S, SC R	hydroxy-sycophantine [21]
**22**	25.67	1105.54	1065.54, 741.44, 579.39	-2Hex, -Hex	SN(B) L, SR S	
**23**	25.67	1046.54	900.47, 754.42, 592.37, 534.76, 430.32	-2DeoxyH, -Hex, -Hex	SQ R, SQ S	
**24**	25.68	1195.54	915.43, 739.41, 577.36		SN(B) L, Sme L, SR L	
**25**	25.73	915.44	753.38		SMe R, SMe S, Sme L	
**26**	25.72	954.5014	808.4436, 722.4440, 576.3874, 488.4536, 414.3350	-DeoxyH, -DeoxyH, -Hex	SN(OB) R, SN(OB) S, SR R, SS S, SS L, SQ R, SQ S, SQ L, SC R, SC S, SC L, SMu R, Smu S, Smu L	malonyl-solamargine [20,28]
**27**	25.77	1065.54	915.46		SN(B) L, SMe L, SR R	
**28**	26.11	1197.59	1035.54, 741.45, 579.39	-Hex, -Pen + Hex, -Hex	SN(OB) R, SN(OB) S, SN(OB) L, SN(B) R, SN(B) S, SN(B) L, SMe S, Sme L, SR R, SR S, SR L	solanigroside E [20,28]
**29**	26.35	884.50	738.44, 592.38, 453.74, 430.33	-DeoxyH, -DeoxyH, -Hex	SS S, SQ S	hydroxy-solamargine [17,18,19]
**30**	26.37	1313.63	1181.59, 887.49, 741.44, 579.39	-Pen, -Pen + Hex, -DeoxyH, -Hex	SN(OB) R, SN(B) R, SR R	
**31**	26.44	1047.53	901.47, 739.42, 577.37	-DeoxyH, -Hex, -Hex	SQ R	
**32**	26.64	1031.54	885.48, 739.42, 577.37, 544.25	-DeoxyH, -DeoxyH, -Hex	SS R, SS S, SS L, SQ R	
**33**	26.56	1089.54	1049.55, 903.49, 741.44, 579.39	-DeoxyH + Hex, -Hex	SMe R, SMe S	
**34**	26.60	1104.46	883.77, 736.64		SN(B) R, SR R, SMu R	
**35**	26.62	1049.55	741.44, 579.39	-DeoxyH, -Hex, -Hex	SMe R, SMe S	anguivioside XI [29,30,31]
**36**	26.91	1033.55	887.50, 741.44, 579.39, 472.23	-DeoxyH, -DeoxyH, -Hex	SS R, SS S	
**37**	26.90	1090.58	766.48, 620.42, 556.79, 458.36	-DeoxyH, -Hex	SMu R	
**38**	27.49	1489.67	1211.57, 887.50, 741.44, 579.39	-Pen + DeoxyH, -Hex + Hex,-DeoxyH, -Hex	SN(B) R, SMe R, SR R	
**39**	27.14	954.51	808.45, 662.39, 488.75, 414.34	-DeoxyH, DeoxyH	SN(OB) R, SN(OB) S, SR R, SS S, SQ R, SQ S, SQ L, SC S, Smu S	
**40**	27.30	885.49	739.42, 577.37	-DeoxyH, -Hex	SS R, SQ S	lyconoside II [32]
**41**	27.41	910.49	764.43, 722.43, 576.37, 466.74, 414.32	-DeoxyH, -DeoxyH, -Hex	SS L, SQ R	
**42**	27.73	1117.54	1045.52, 739.42		SS L	
**43**	27.83	1181.59	887.50, 741.39, 579.39	-Pen + Hex, -DeoxyH, -Hex	SN(B) R	solanigroside D [27]
**44**	27.99	1563.75	793.37, 753.38, 607.33, 445.28		SN(B) S	
**45**	28.09	1073.53	910.49		SQ R	
**46**	28.50	1313.64	887.50, 741.44, 579.39	-Hex + 2Pen, -DeoxyH, -Hex	SN(OB) R, SN(OB) S, SN(OB) L, SN(B) R, SMe R, SMe S, SR R	
**47**	28.65	1313.64	1181.60, 887.50, 741.45, 579.39	-Pen, -Pen + Hex, -DeoxyH, -Hex	SN(OB) R, SN(OB) S, SN(OB) L, SN(B) R, SMe R, SMe S, SR R	
**48**	29.32	926.49	738.42, 474.74		SQ R	
**49**	29.40	1085.5160	901.4786, 755.4214, 593.3703, 431.3170	-Hex	SS R	
**50**	29.87	1069.5218	885.4863, 739.4280, 593.3703, 431.3170	-DeoxyH, -DeoxyH, -Hex	SS R	
**51**	37.12	1149.57	1017.53, 871.47, 723.43, 577.38, 415.32	-Pen, -DeoxyH, -Pen + 2Hex, -Hex	SN(OB) S, SN(OB) L	

* Compound numbers refer to the corresponding compound numbers mentioned in text.

**Table 2:** Pearson’s correlations for tested *Solanum* plant extracts based on their glycoalkaloid composition and content. * *p* < 0.05, ** *p* < 0.01, *** *p* < 0.001.

	*S. nigrum (OB)* Roots	Stems	Leaves	*S. nigrum (B)* Roots	Stems	Leaves	*S. melanocerasum* Roots	Stems	Leaves	*S. retroflexum* Roots	Stems	Leaves	*S. sisymbriifolium* Roots	Stems	Leaves	*S. quitoense* Roots	Stems	Leaves	*S. caripense * Roots	Stems	Leaves	*S. muricatum* Roots	Stems
***S. nigrum*(OB)** Stems	0.526 ***																						
Leaves	0.311 *	0.951 ***																					
***S. nigrum (B)*** Roots	0.524 ***	0.508 ***	0.528 ***																				
Stems	0.243	0.825 ***	0.920 ***	0.460 ***																			
Leaves	0.188	0.816 ***	0.898 ***	0.276	0.969 ***																		
***S. melanocerasum*** Roots	0.124	0.166	0.192	0.332 *	0.141	0.072																	
Stems	0.093	0.416 **	0.471 ***	0.224	0.494 ***	0.482 ***	0.878 ***																
Leaves	0.159	0.782 ***	0.853 ***	0.196	0.933 ***	0.979 ***	0.158	0.574 ***															
***S. retroflexum*** Roots	0.618 ***	0.770 ***	0.705 ***	0.679 ***	0.495 ***	0.445 **	0.287 *	0.283 *	0.396 **														
Stems	0.282 *	0.830 ***	0.917 ***	0.497 ***	0.997 ***	0.957 ***	0.149	0.491 ***	0.922 ***	0.503 ***													
Leaves	0.214	0.834 ***	0.903 ***	0.294 *	0.971 ***	0.994 ***	0.080	0.486 ***	0.984 ***	0.466 ***	0.962 ***												
***S. sisymbriifolium*** Roots	−0.054	−0.098	−0.107	−0.080	−0.048	−0.071	−0.086	−0.081	−0.069	−0.106	−0.079	−0.068											
Stems	−0.001	−0.093	−0.121	−0.103	−0.058	−0.082	−0.098	−0.096	−0.079	−0.100	−0.086	−0.076	0.658 ***										
Leaves	0.454 ***	0.069	−0.071	−0.049	−0.041	−0.057	−0.053	−0.069	−0.056	0.087	−0.041	−0.046	0.159	0.319 *									
***S. quitoense*** Roots	0.032	−0.103	−0.135	−0.108	−0.100	−0.095	−0.110	−0.107	−0.09	−0.103	−0.098	−0.086	0.075	0.090	0.063								
Stems	0.787 ***	0.157	−0.080	0.031	−0.066	−0.071	−0.058	−0.098	−0.069	0.184	−0.038	−0.052	0.051	0.205	0.609 ***	0.256							
Leaves	0.872 ***	0.197	−0.062	0.148	−0.057	−0.061	−0.045	−0.082	−0.058	0.219	−0.022	−0.039	0.027	0.115	0.534 ***	0.244	0.922 ***						
***S. caripense*** Roots	−0.001	−0.063	−0.085	−0.047	−0.022	−0.056	−0.069	−0.078	−0.056	−0.073	−0.063	−0.054	0.727 ***	0.718 ***	0.225	0.089	0.132	0.095					
Stems	0.661 ***	0.126	−0.077	−0.001	−0.043	−0.065	−0.056	−0.093	−0.065	0.147	−0.041	−0.051	0.369 **	0.441 **	0.625 ***	0.109	0.834 ***	0.772 ***	0.551 ***				
Leaves	0.597 ***	0.137	−0.037	0.038	−0.024	−0.037	−0.025	−0.053	−0.037	0.158	−0.013	−0.025	0.029	0.100	0.907 ***	0.074	0.683 ***	0.654 ***	0.100	0.629 ***			
***S. muricatum*** Roots	0.065	−0.050	−0.091	−0.044	−0.026	−0.061	−0.073	−0.082	−0.060	−0.056	−0.065	−0.058	0.702 ***	0.688 ***	0.334 *	0.037	0.168	0.150	0.966 ***	0.614 ***	0.225		
Stems	0.161	0.001	−0.062	−0.022	−0.007	−0.043	−0.046	−0.061	−0.044	−0.001	−0.042	−0.040	0.715 ***	0.697 ***	0.394 **	0.059	0.285 *	0.245	0.953 ***	0.694 ***	0.284 *	0.960 ***	
Leaves	0.635 ***	0.150	−0.034	0.071	−0.023	−0.037	−0.023	−0.052	−0.036	0.171	−0.01	−0.023	0.039	0.099	0.894 ***	0.079	0.694 ***	0.695 ***	0.116	0.634 ***	0.992 ***	0.241	0.297 *

**Table 3:** Czekanowski similarity coefficients for tested *Solanum* plant extracts based on their glycoalkaloid composition and content.

	*S. nigrum (OB)* Roots	Stems	Leaves	*S. nigrum (B)* Roots	Stems	Leaves	*S. melanocerasum* Roots	Stems	Leaves	*S. retroflexum* Roots	Stems	Leaves	*S. sisymbriifolium* Roots	Stems	Leaves	*S. quitoense* Roots	Stems	Leaves	*S. caripense* Roots	Stems	Leaves	*S. muricatum* Roots	Stems	Leaves
***S. nigrum (OB)*** Roots	1.00																							
Stems	0.32	1.00																						
Leaves	0.42	0.48	1.00																					
***S. nigrum (B)*** Roots	0.09	0.36	0.15	1.00																				
Stems	0.06	0.24	0.10	0.39	1.00																			
Leaves	0.10	0.37	0.18	0.28	0.65	1.00																		
***S. melanocerasum*** Roots	0.08	0.26	0.13	0.54	0.25	0.20	1.00																	
Stems	0.09	0.29	0.14	0.34	0.48	0.47	0.73	1.00																
Leaves	0.09	0.33	0.16	0.18	0.53	0.83	0.19	0.46	1.00															
***S. retroflexum*** Roots	0.31	0.70	0.42	0.39	0.27	0.36	0.30	0.33	0.31	1.00														
Stems	0.28	0.56	0.55	0.32	0.24	0.41	0.23	0.30	0.35	0.39	1.00													
Leaves	0.23	0.46	0.68	0.24	0.18	0.31	0.19	0.23	0.28	0.31	0.74	1.00												
***S. sisymbriifolium*** Roots	0.01	0.02	0.01	0.05	0.08	0.02	0.03	0.05	0.01	0.03	0.02	0.01	1.00											
Stems	0.02	0.06	0.03	0.05	0.04	0.01	0.04	0.06	0.01	0.07	0.07	0.03	0.17	1.00										
Leaves	0.15	0.09	0.02	0.02	0.02	0.01	0.02	0.05	0.00	0.10	0.04	0.02	0.09	0.42	1.00									
***S. quitoense*** Roots	0.04	0.11	0.05	0.07	0.03	0.01	0.03	0.04	0.01	0.12	0.08	0.04	0.09	0.27	0.17	1.00								
Stems	0.06	0.21	0.05	0.15	0.05	0.02	0.05	0.02	0.01	0.23	0.10	0.05	0.15	0.26	0.18	0.28	1.00							
Leaves	0.01	0.04	0.02	0.13	0.05	0.02	0.07	0.01	0.01	0.05	0.04	0.03	0.16	0.09	0.03	0.06	0.23	1.00						
***S. caripense*** Roots	0.04	0.09	0.04	0.08	0.02	0.01	0.04	0.03	0.01	0.10	0.06	0.04	0.08	0.32	0.20	0.25	0.22	0.05	1.00					
Stems	0.23	0.19	0.04	0.07	0.02	0.01	0.02	0.02	0.00	0.19	0.06	0.03	0.06	0.22	0.43	0.15	0.22	0.04	0.49	1.00				
Leaves	0.38	0.10	0.03	0.04	0.01	0.00	0.01	0.01	0.00	0.09	0.03	0.02	0.03	0.13	0.34	0.08	0.08	0.02	0.13	0.31	1.00			
***S. muricatum*** Roots	0.01	0.05	0.02	0.10	0.09	0.03	0.05	0.05	0.02	0.06	0.05	0.03	0.29	0.40	0.22	0.13	0.21	0.19	0.28	0.22	0.10	1.00		
Stems	0.02	0.10	0.03	0.09	0.06	0.02	0.05	0.01	0.01	0.10	0.06	0.04	0.18	0.46	0.24	0.17	0.33	0.19	0.36	0.29	0.12	0.63	1.00	
Leaves	0.27	0.16	0.03	0.09	0.02	0.01	0.02	0.00	0.00	0.15	0.05	0.03	0.06	0.12	0.52	0.11	0.17	0.04	0.15	0.44	0.54	0.15	0.18	1.00

**Table 4:** AChE inhibition of *Solanum* plants root, stem, and leaf extracts expressed as percentage, and the half-maximal inhibitory concentration (IC_50_) relative to the total glycoalkaloid and plant mass basis.

Solanum Plant		Inhibition, %	IC_50_, µg Glycoalkaloid mL^−1^	IC_50_, mg Plant mL^−1^
*S. caripense*	Roots	57	94.8	283.4
	Stems	60	120.6	261.7
	Leaves	80	10.5	13.8
*S. melanocerasum*	Roots	48	62.0	438.2
	Stems	49	1.4	10.8
	Leaves	92	0.5	6.9
*S. muricatum*	Roots	70	1.2	20.9
	Stems	46	2.2	32.6
	Leaves	87	6.3	25.4
*S. nigrum (B)*	Roots	58	48.8	577.3
	Stems	39	1.0	19.3
	Leaves	82	0.8	8.8
*S. nigrum (OB)*	Roots	67	344.9	238.1
	Stems	56	28.4	102.3
	Leaves	82	141.0	149.5
*S. quitoense*	Roots	69	0.5	1.8
	Stems	68	0.6	8.8
	Leaves	73	0.4	44.9
*S. retroflexum*	Roots	53	61.8	187.1
	Stems	65	32.2	103.3
	Leaves	84	1.5	3.6
*S. sisymbriifolium*	Roots	77	5.6	302.7
	Stems	83	0.8	6.8
	Leaves	93	0.7	2.5

## Data Availability

The data presented in this study are available on request from the corresponding author.

## Supplementary Materials

The following are available online at https://www.mdpi.com/article/10.3390/plants11030269/s1, Table S1: Concentrations of individual glycoalkaloids in plant extracts analyzed. Figure S1: HPLC TOF MS chromatographic profiles of leaves, stems, and roots of studied *Solanum* plants. Figure S2. Reported structures of glycoalkaloids referenced in the manuscript and identified in Table 1.
