# Supplementary material for: High-Performance Liquid Chromatography–Mass Spectrometry Analysis of Glycoalkaloids from Underexploited Solanum Species and Their Acetylcholinesterase Inhibition Activity
Source: Plants (Basel). 2022 Jan 20;11(3):269. doi: 10.3390/plants11030269 (PMC8839269; doi:10.3390/plants11030269)

Figure S1. Total ion chromatograms of methanolic extract from the selected *Solanum* species analyzed by HPLC TOF MS. Peak numbers correspond to glycoalkaloid compounds identified in Table 1.

Solanum muricatum

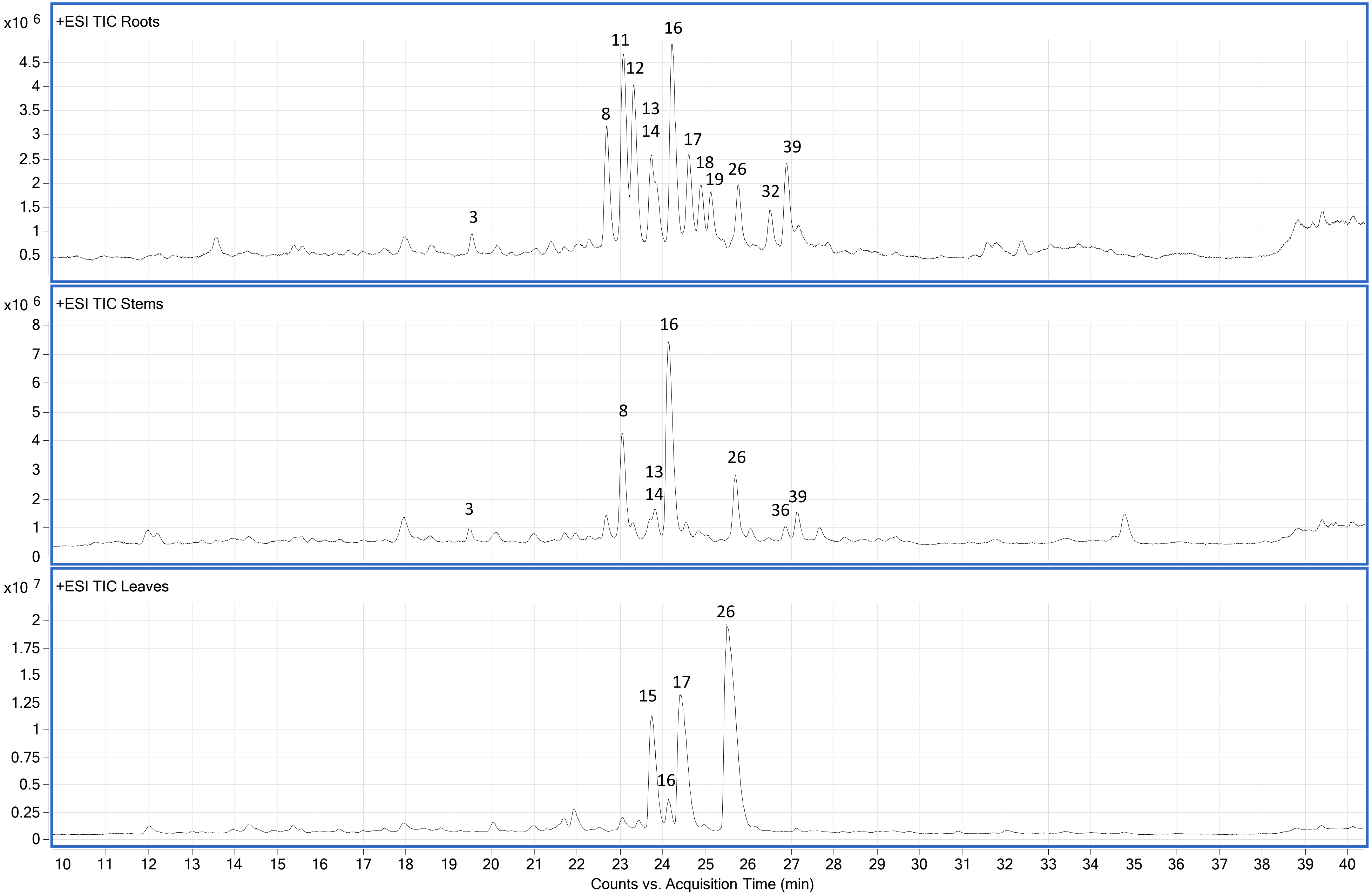

Solanum caripense

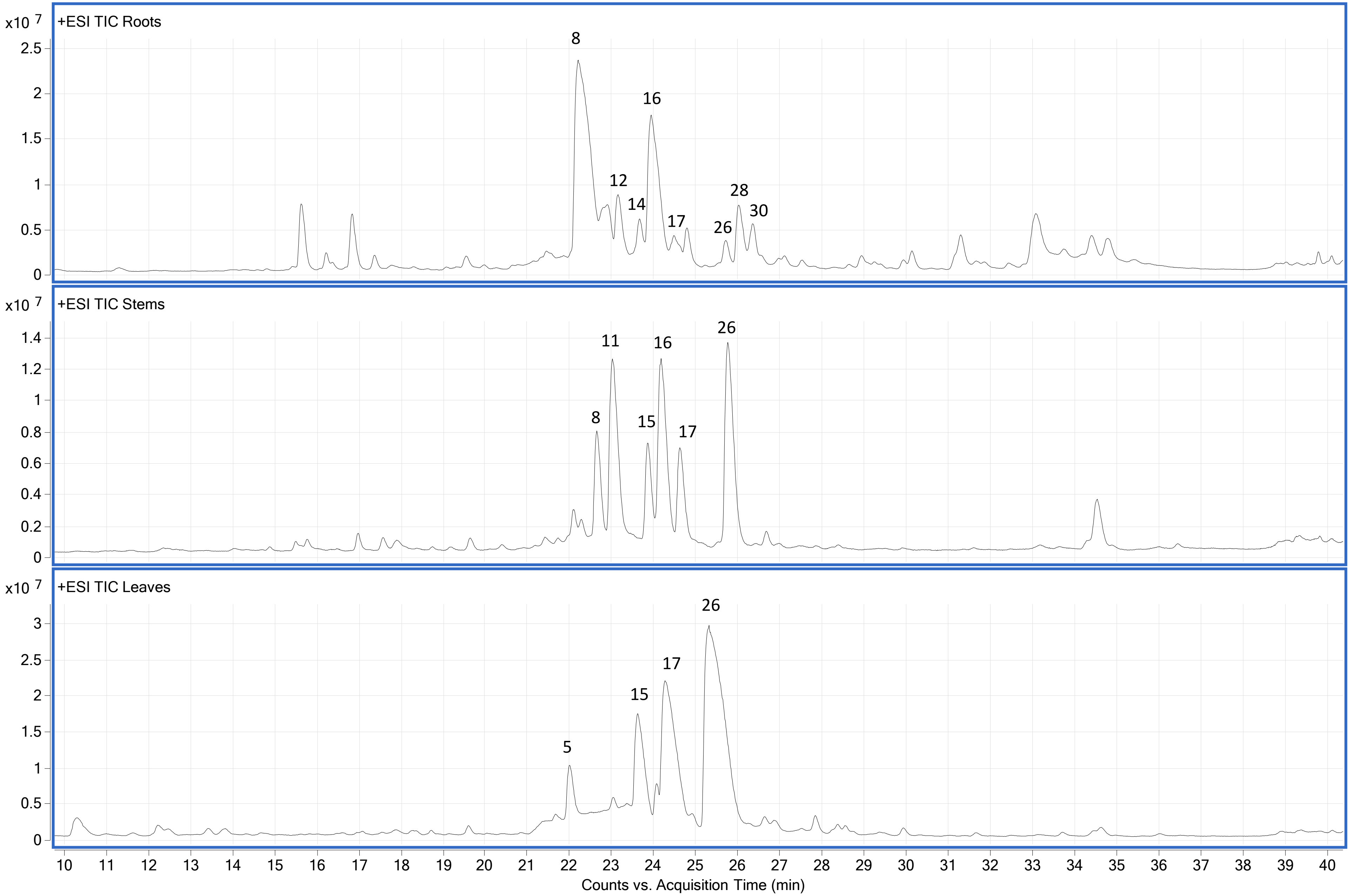

Solanum nigrum (Blackberries)

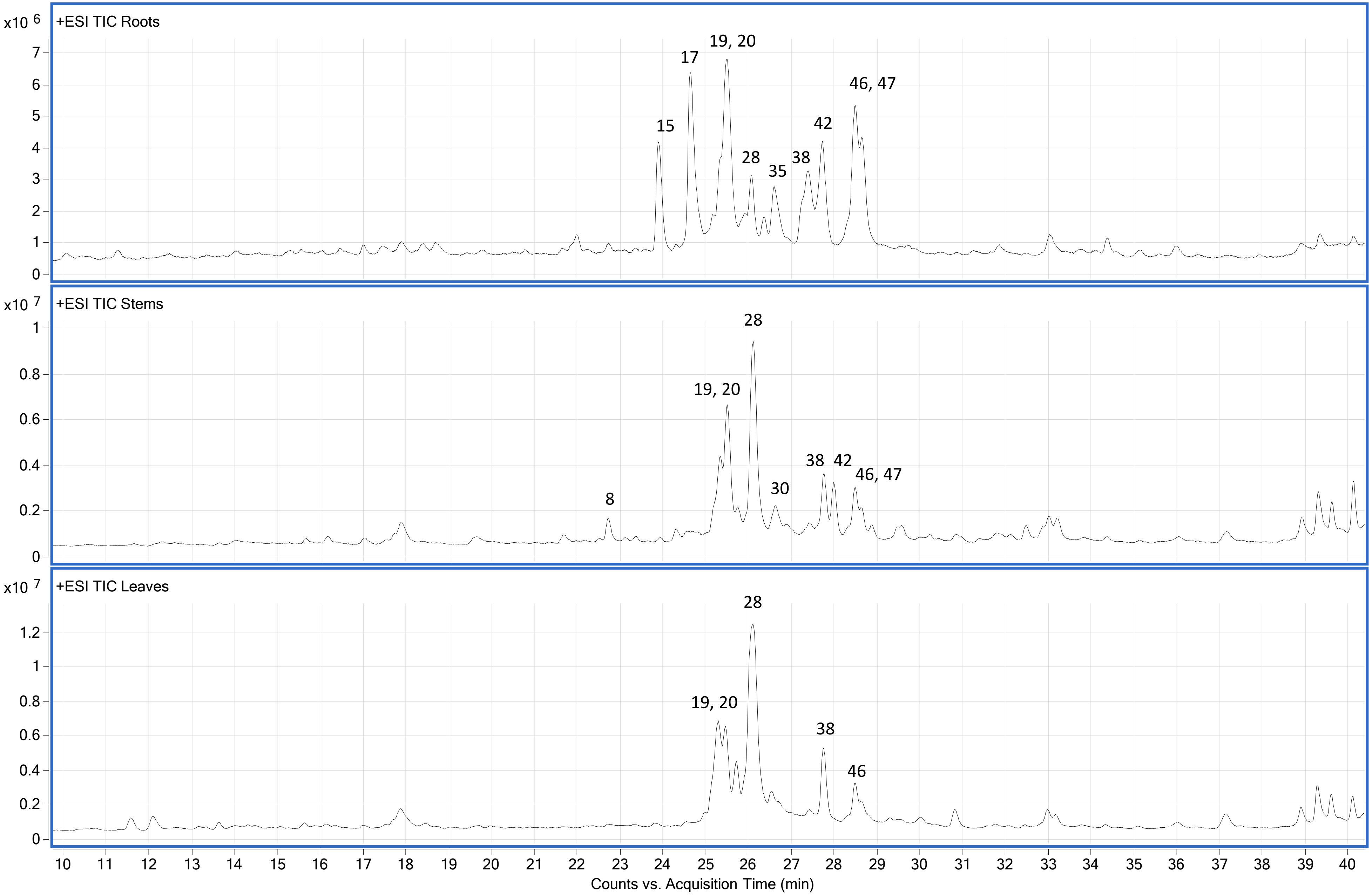

Solanum melanocerasum

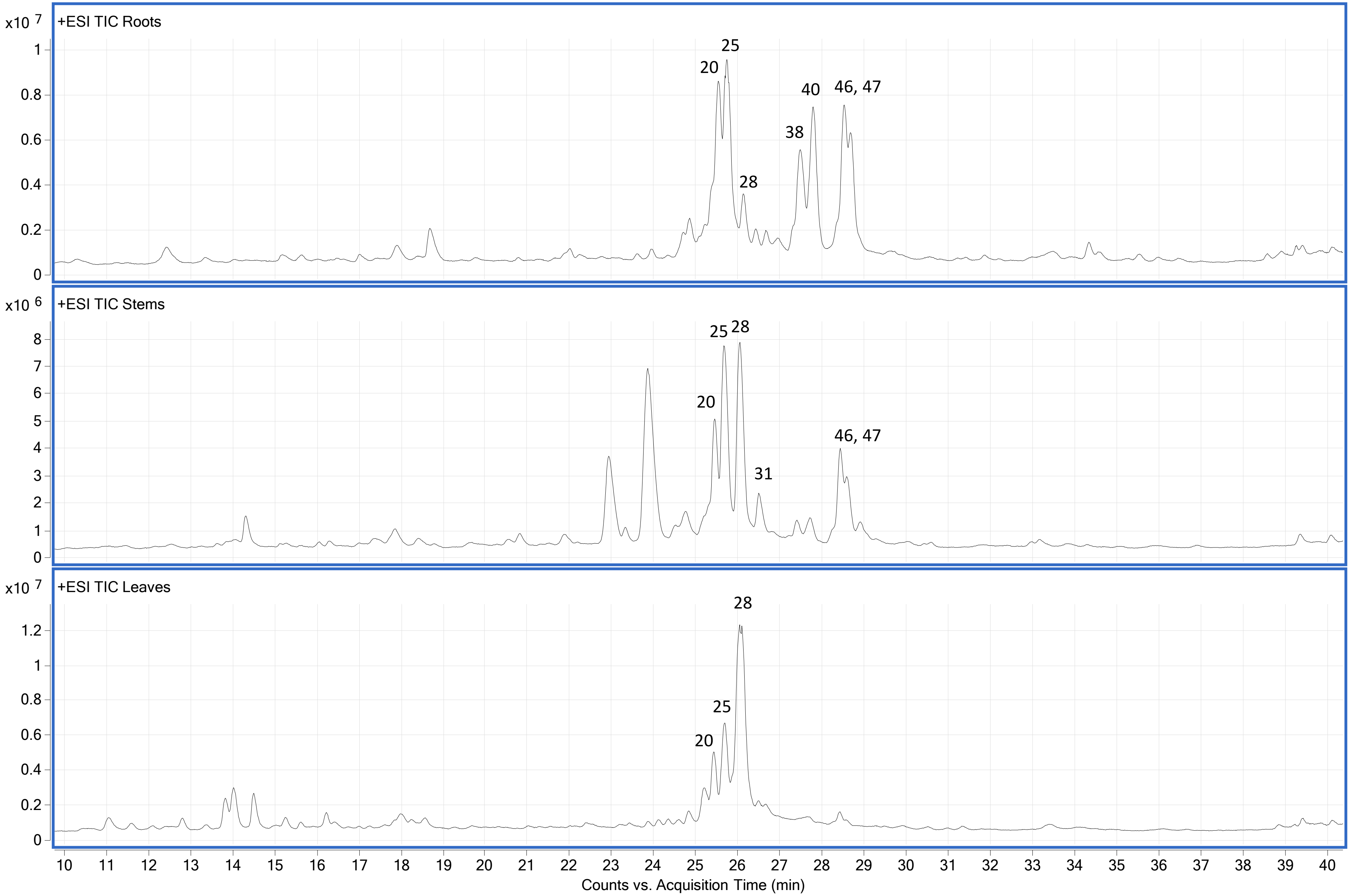

Solanum quitoense

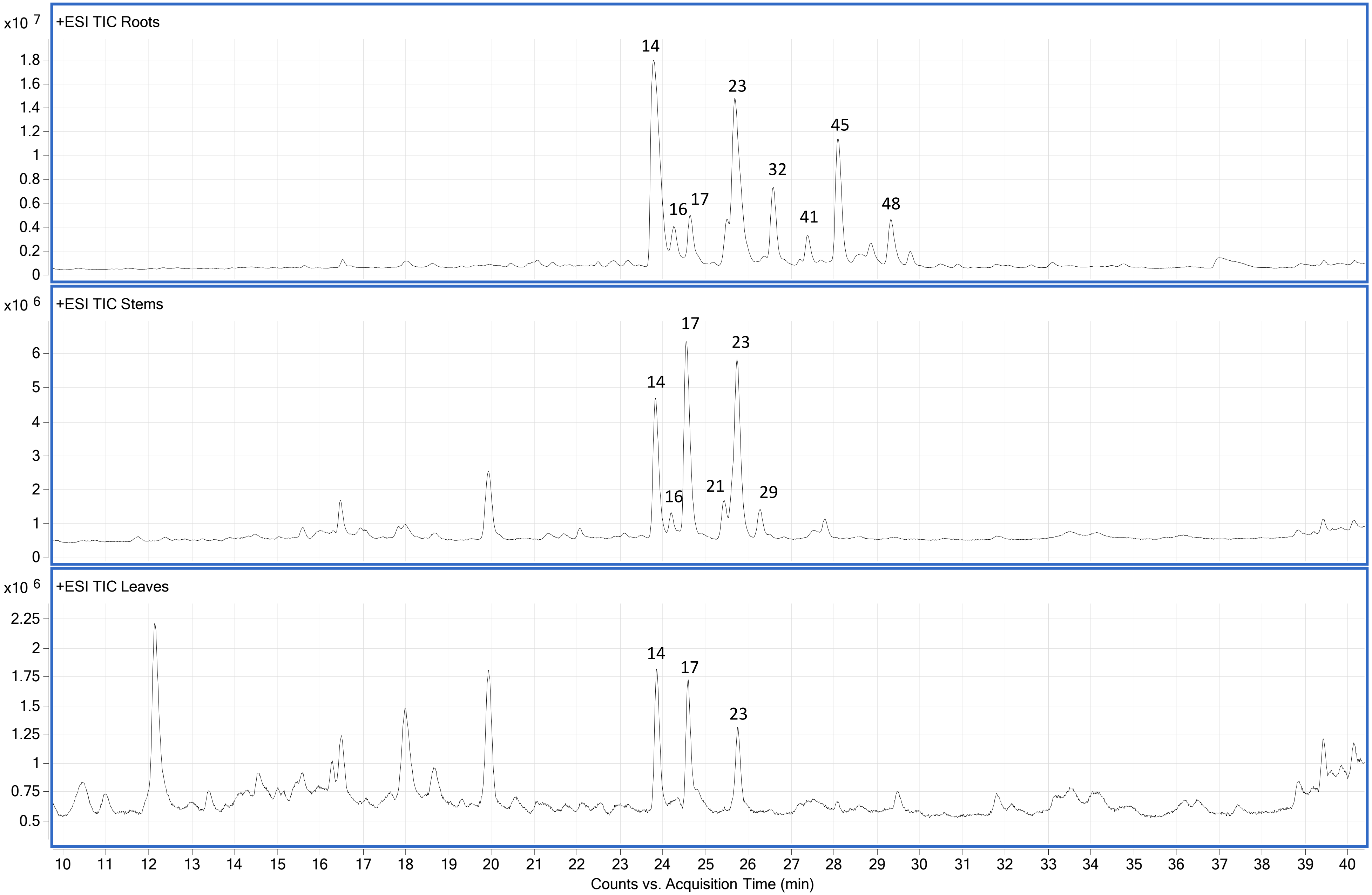

Solanum sysimbriifolium

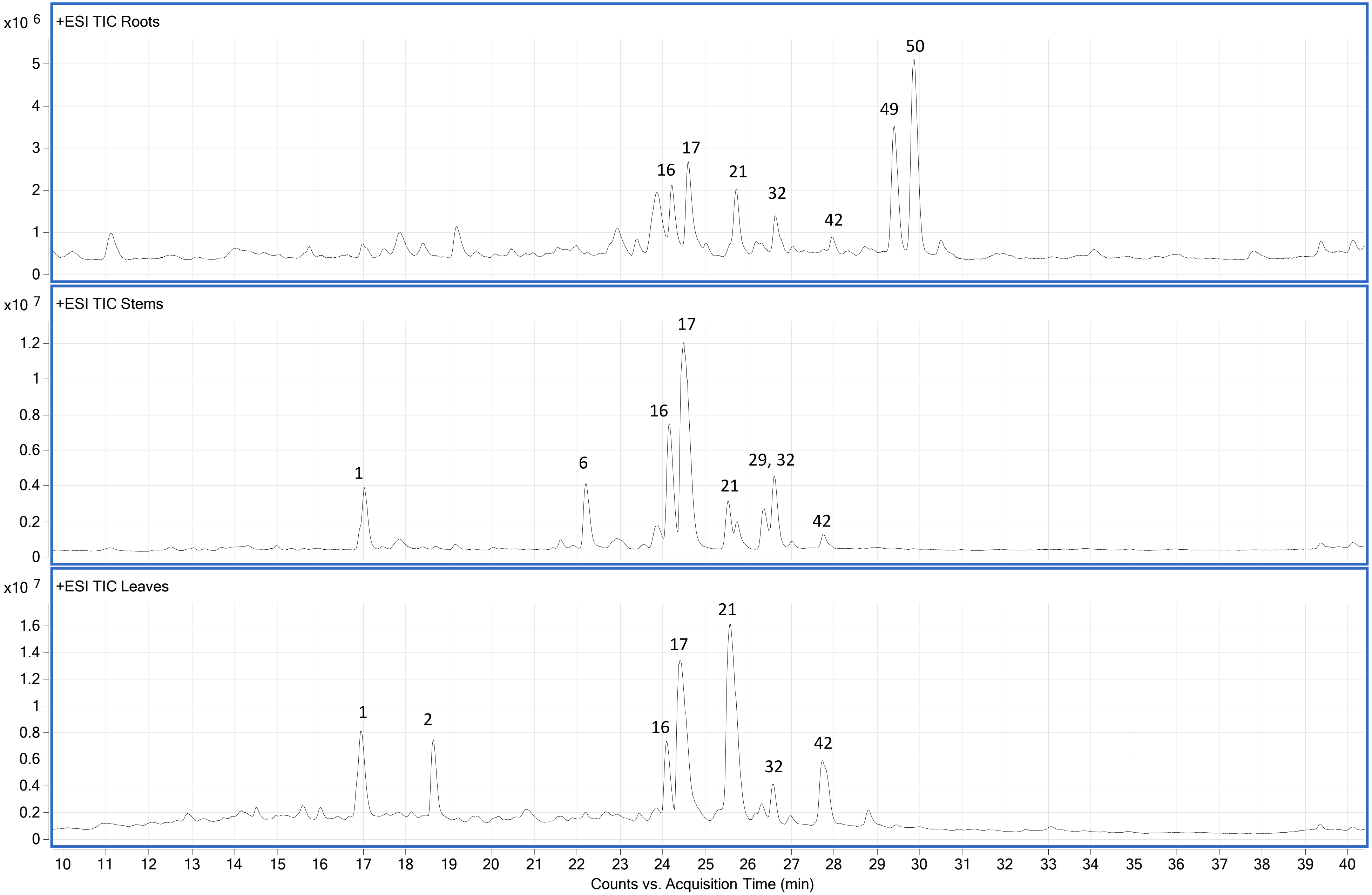

Solanum retroflexum

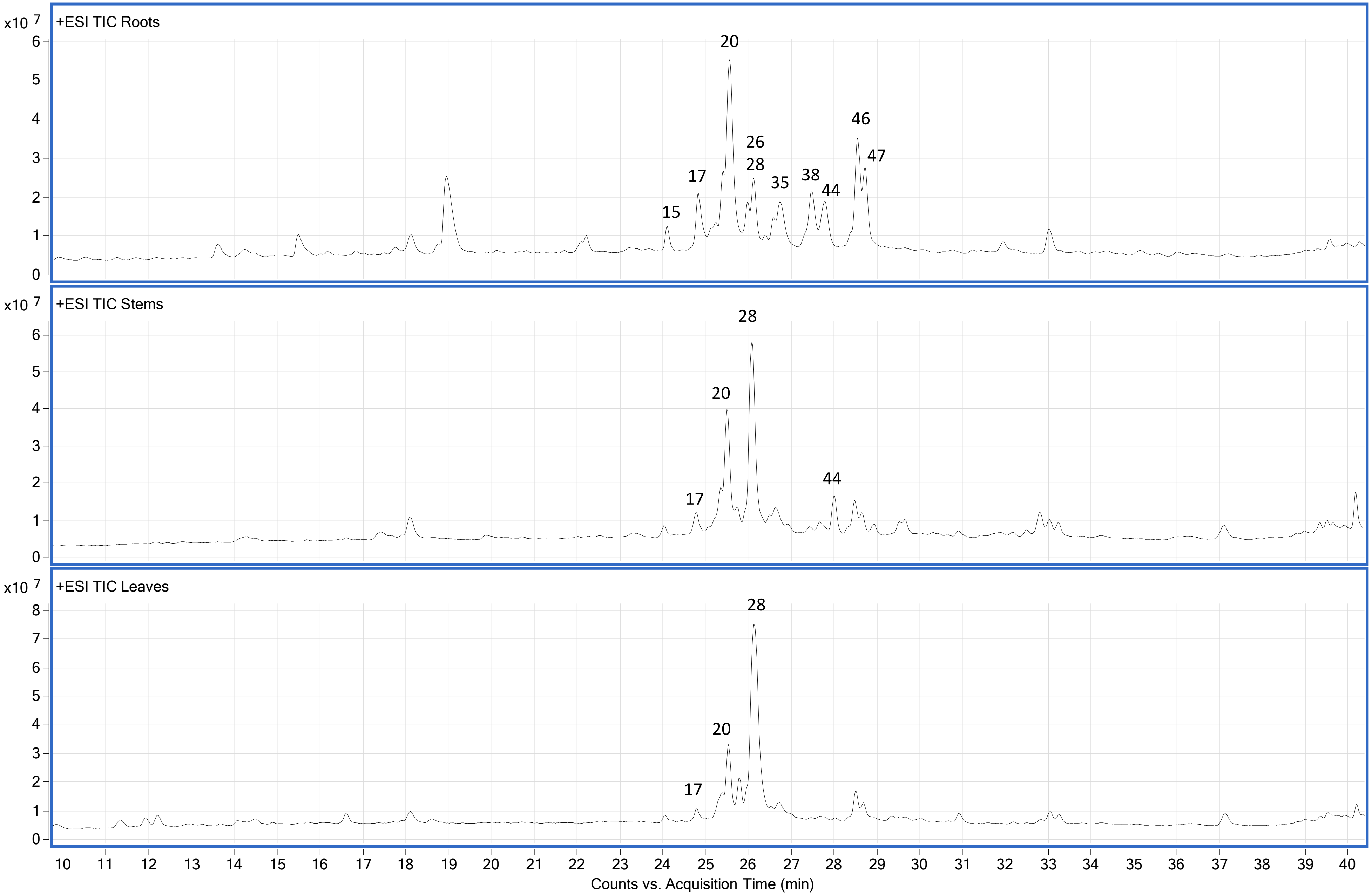

Solanum nigrum (Orange Berry)

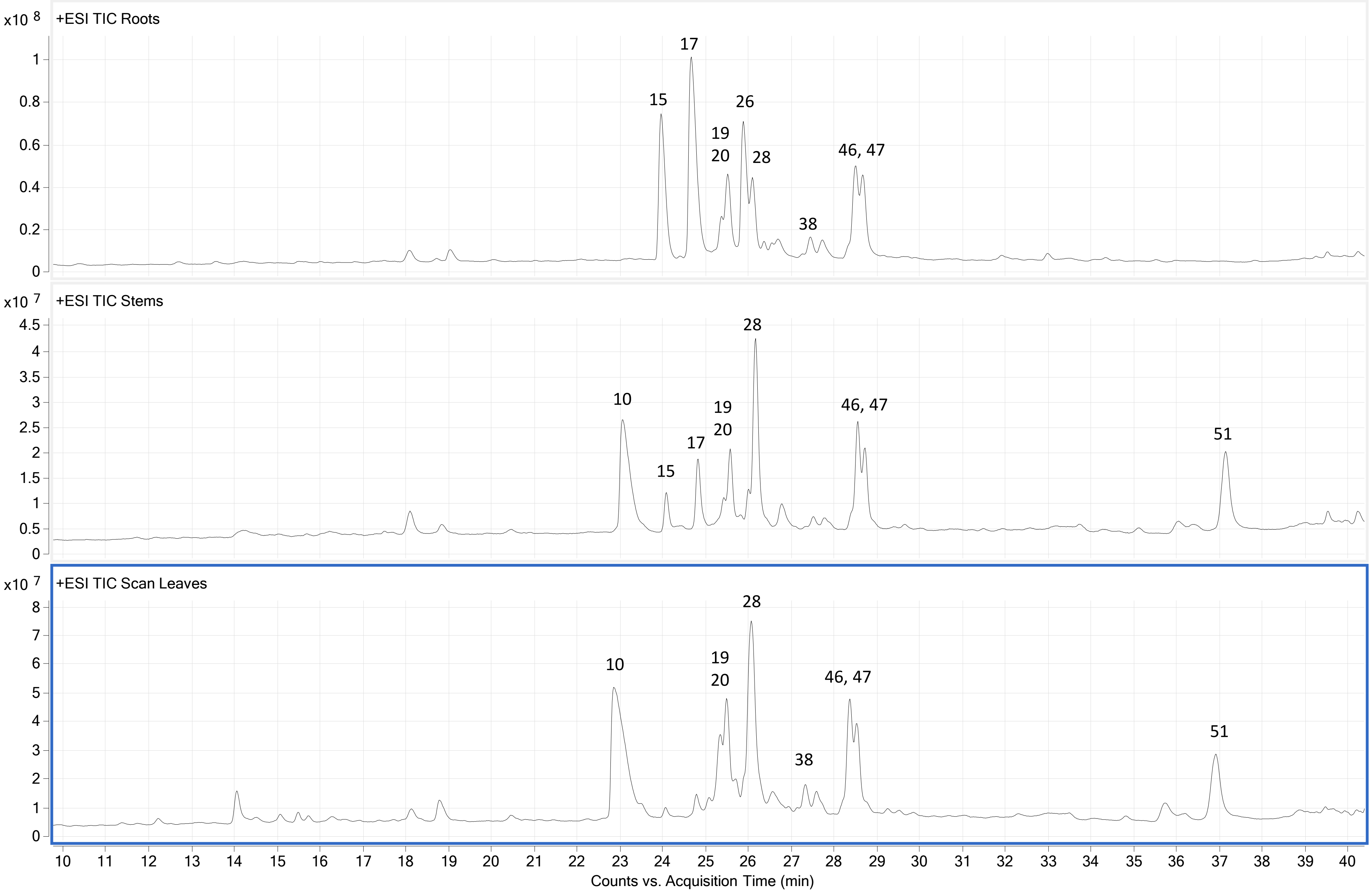

Supplement: Supplementary file 1 [file plants-11-00269-s001.zip › Figure S1.pdf]
