# Supplementary material for: High-Performance Liquid Chromatography–Mass Spectrometry Analysis of Glycoalkaloids from Underexploited Solanum Species and Their Acetylcholinesterase Inhibition Activity
Source: Plants (Basel). 2022 Jan 20;11(3):269. doi: 10.3390/plants11030269 (PMC8839269; doi:10.3390/plants11030269)

Figure S2. Reported structures of glycoalkaloids referenced in the manuscript and identified in Table 1.

Hydroxy-solamargine (***1, 29***)

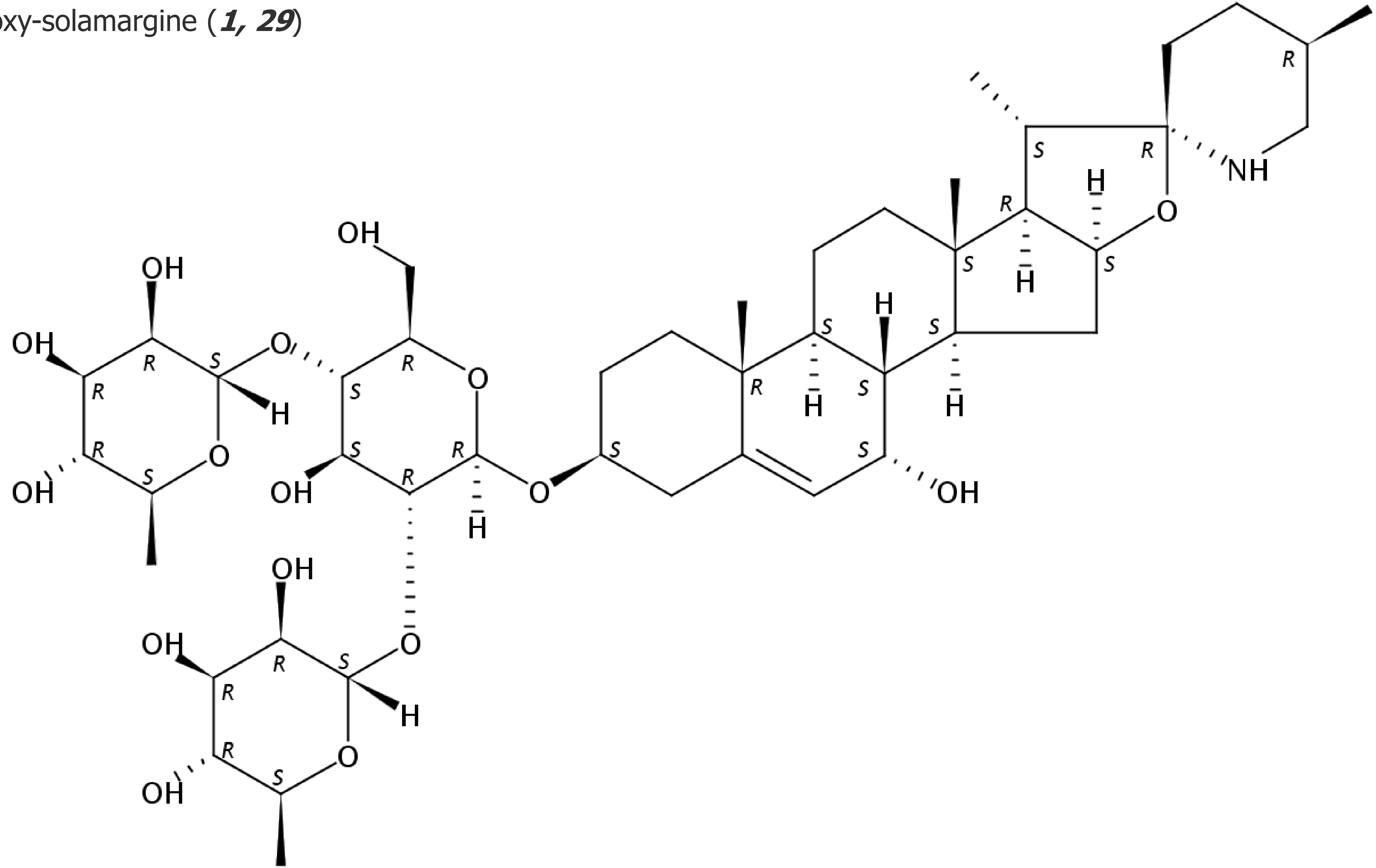

Malonyl-solanandaine (***2***)

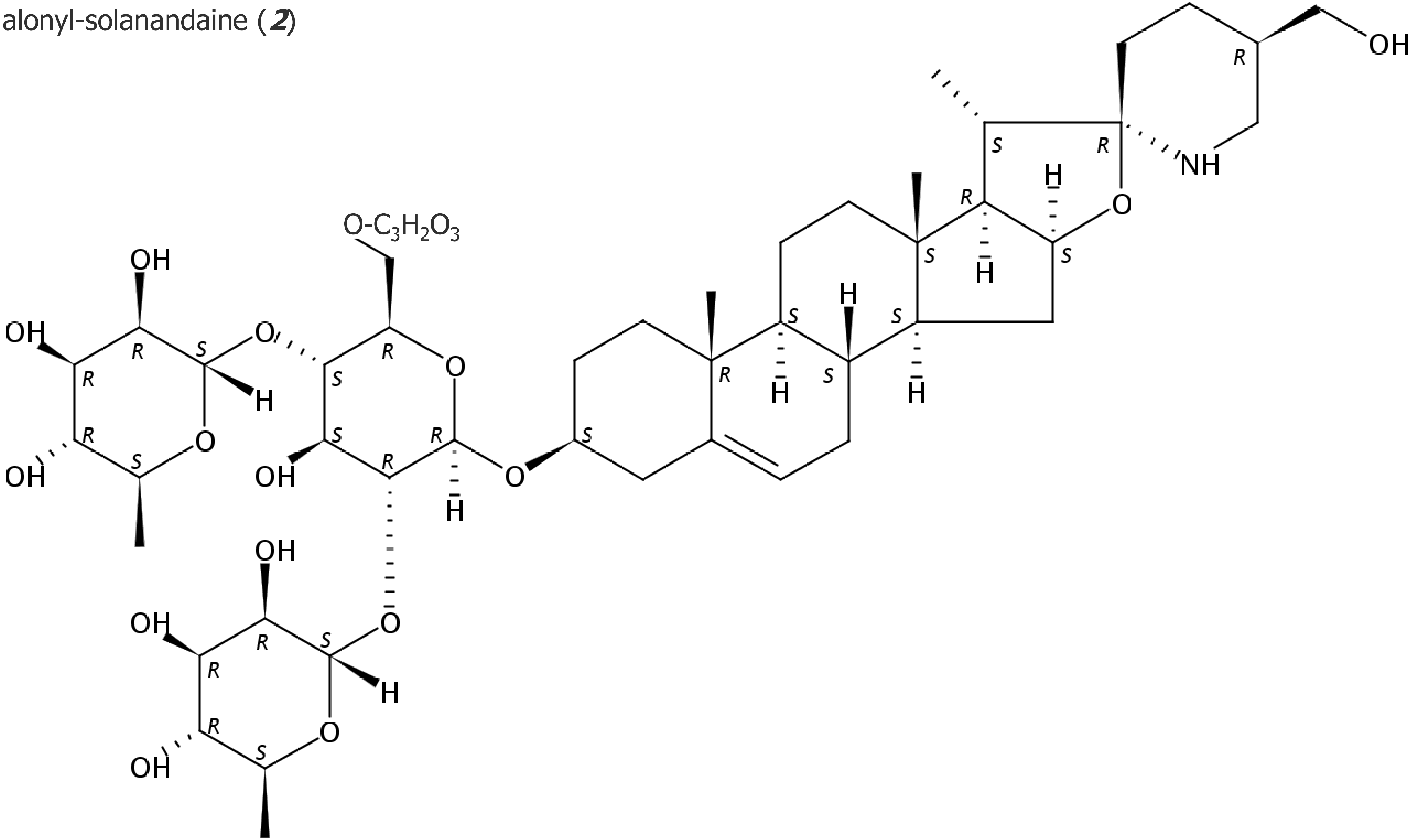

Arudonine (***3***)

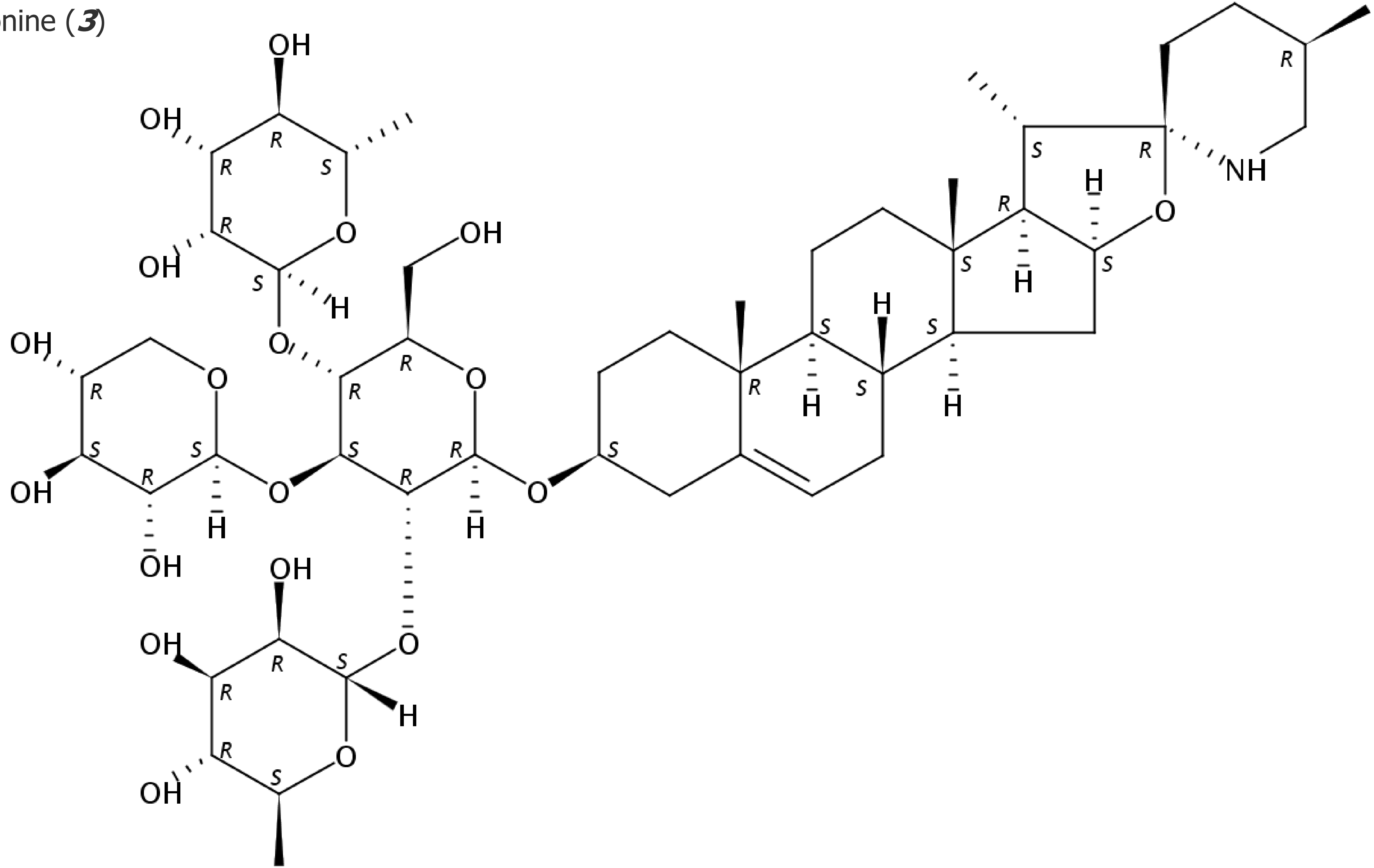

Solamargine (***6, 17***)

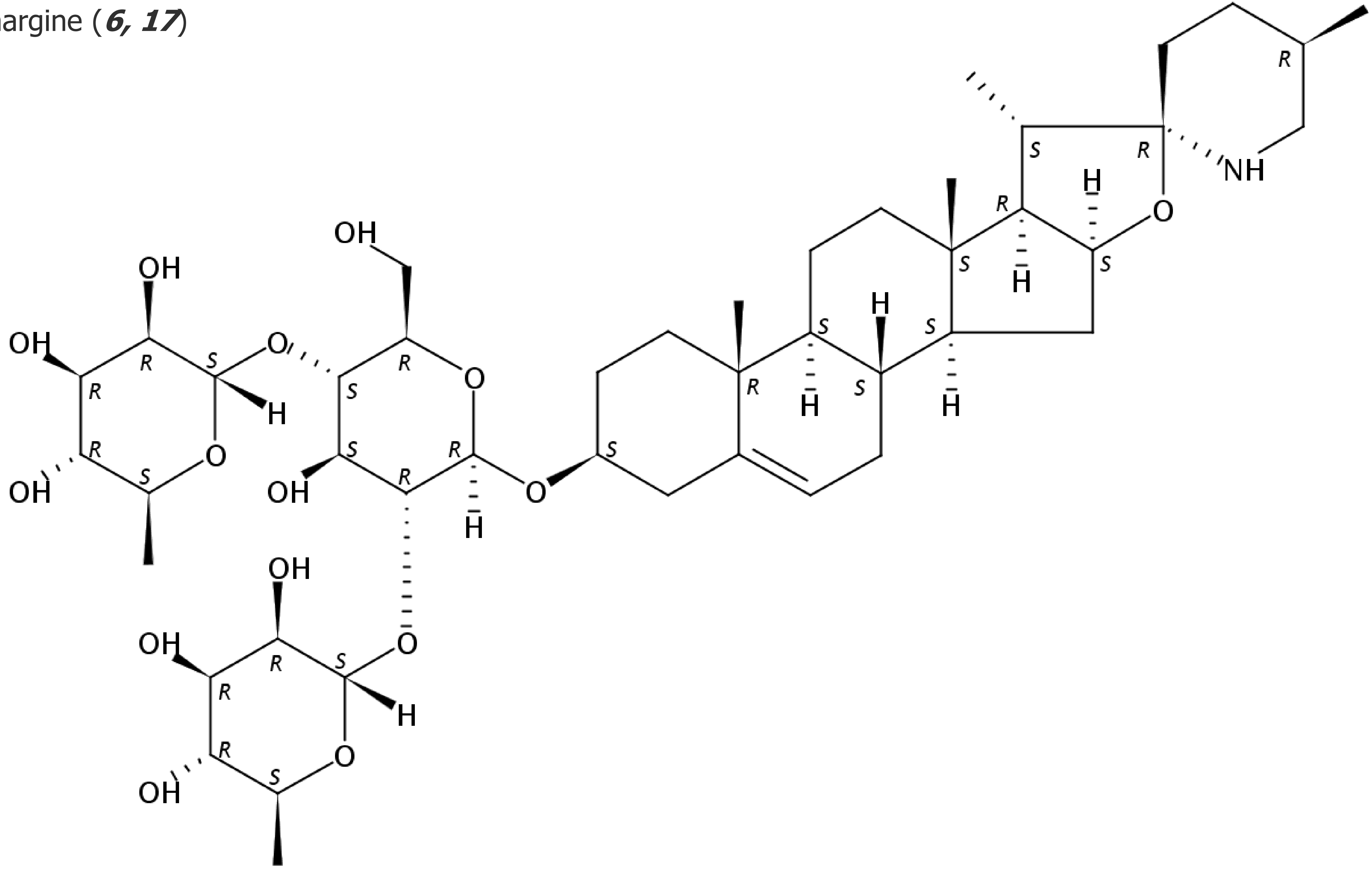

Solaviaside B (7)

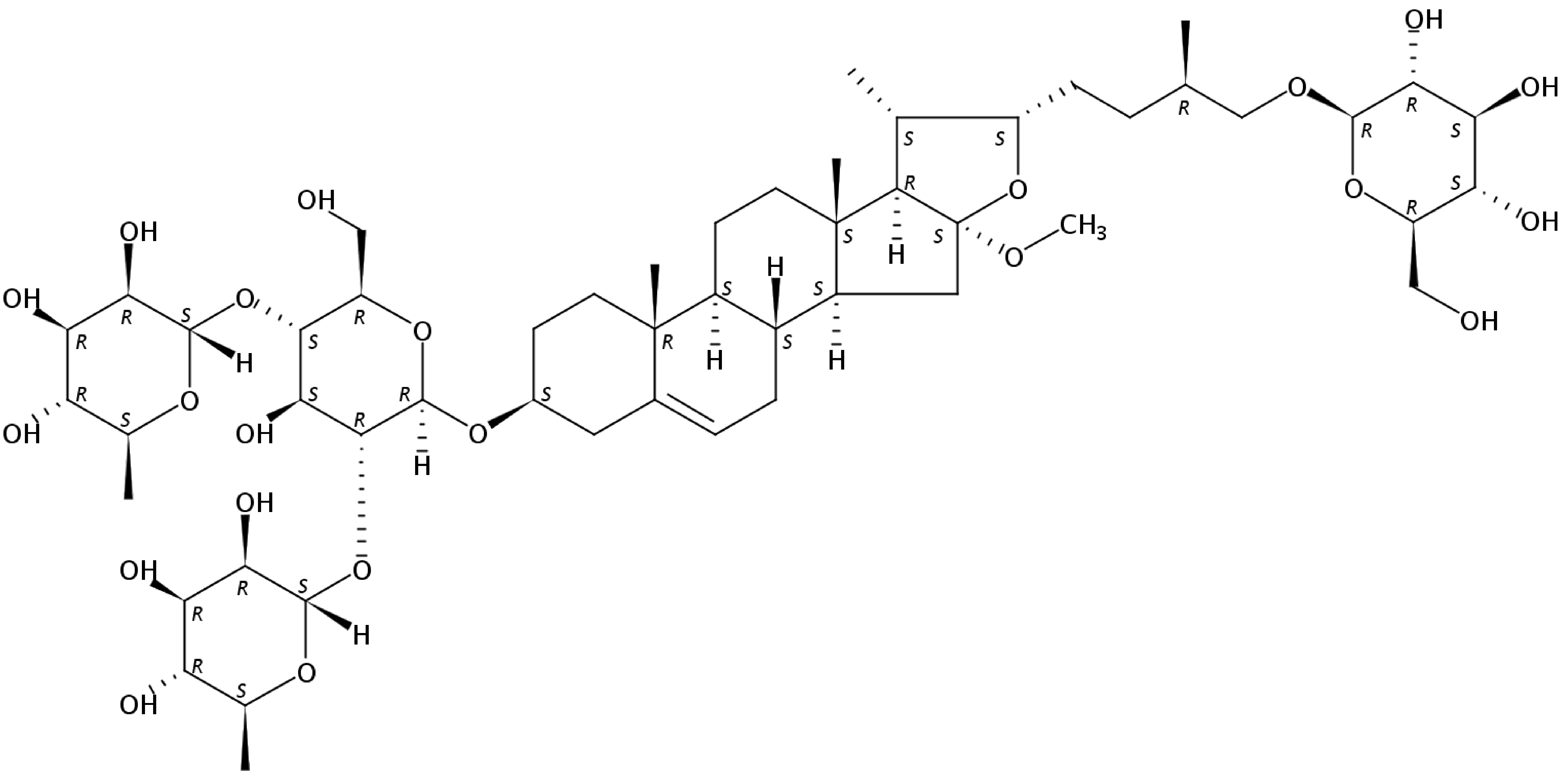

Solaviaside B (*9*)

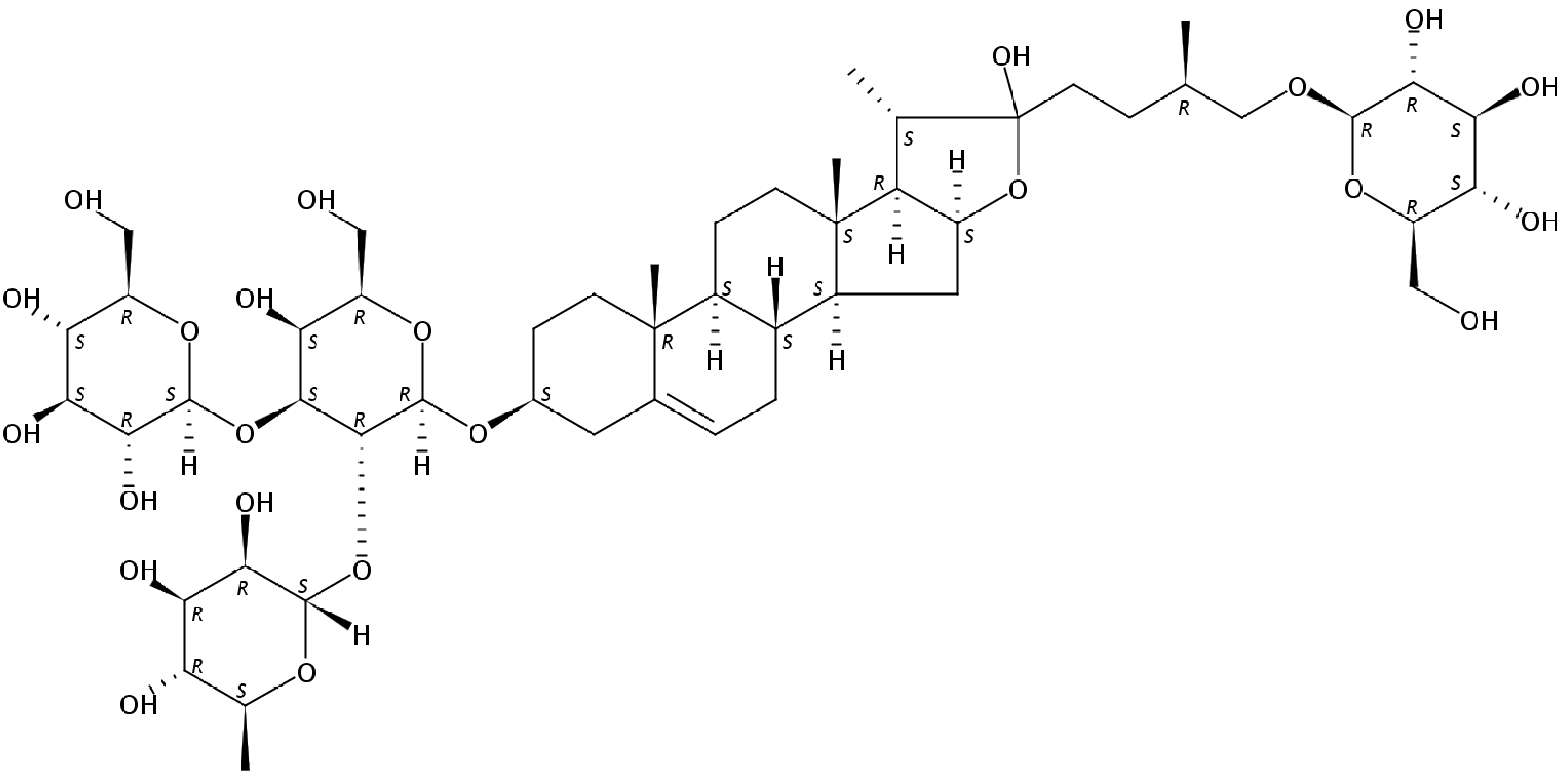

Solanigroside H (***12, 13***)

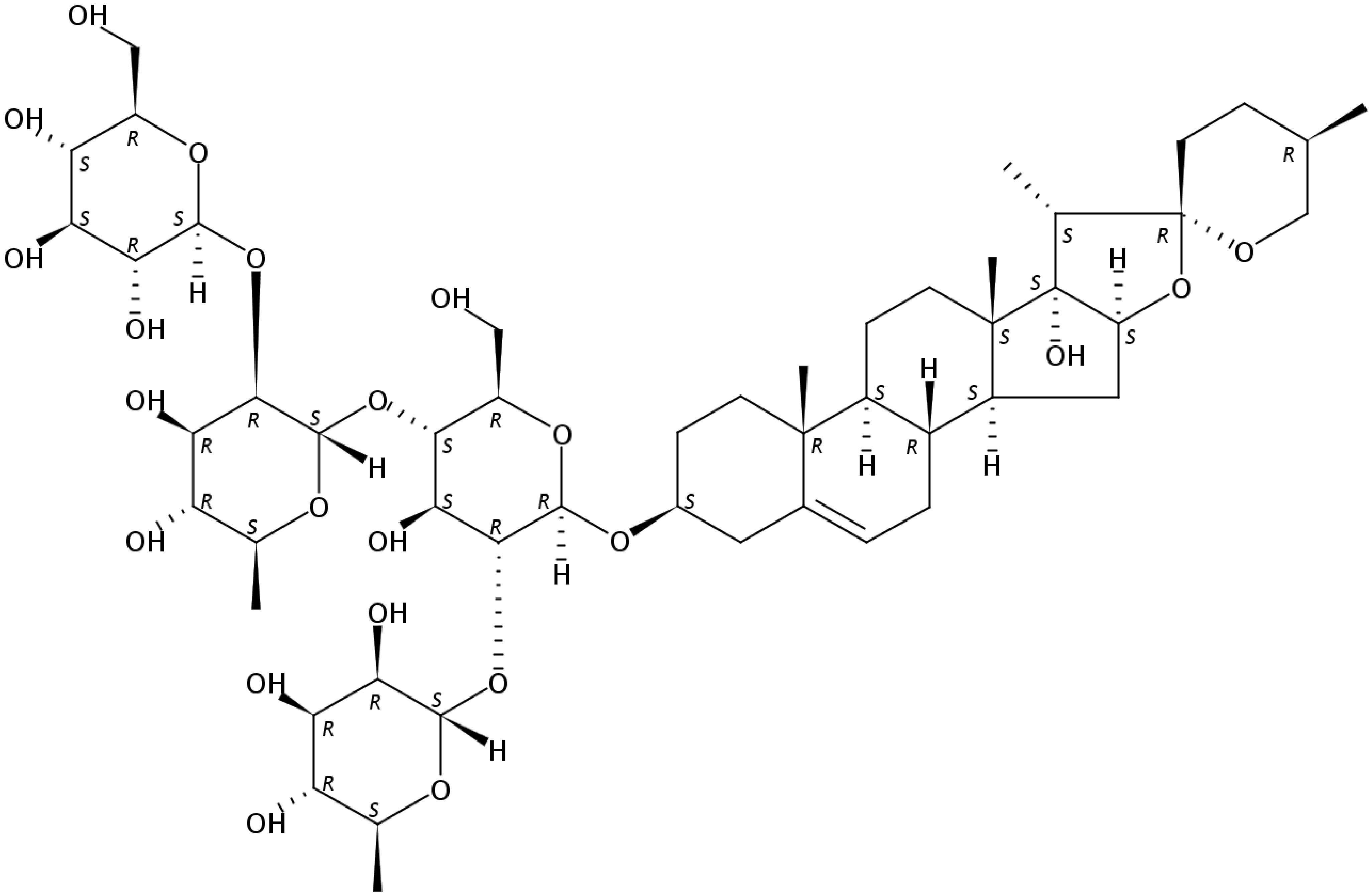

Solamarine (***15***)

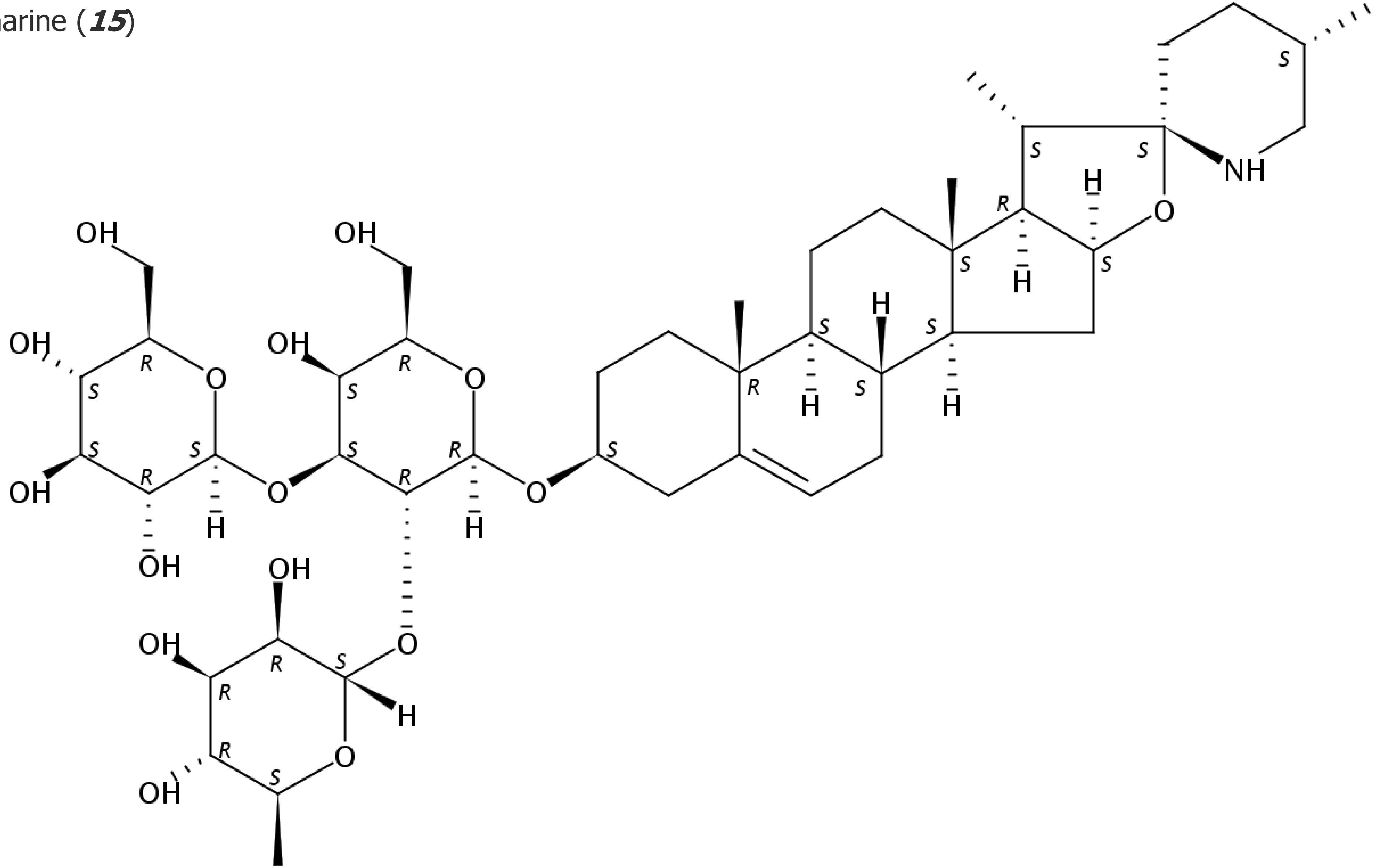

Sycophantine (***16***)

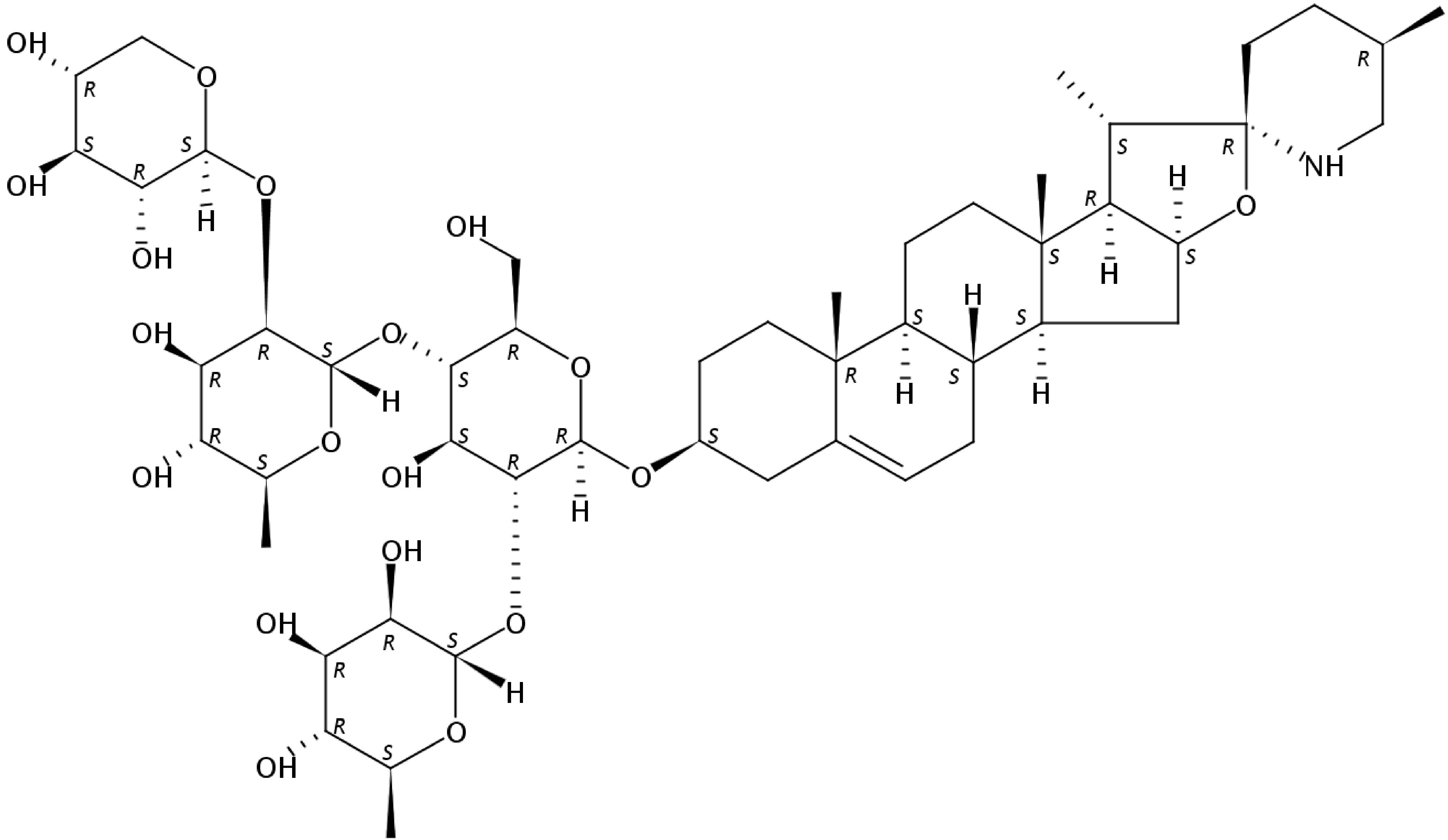

Solanigroside Y5 (***20***)

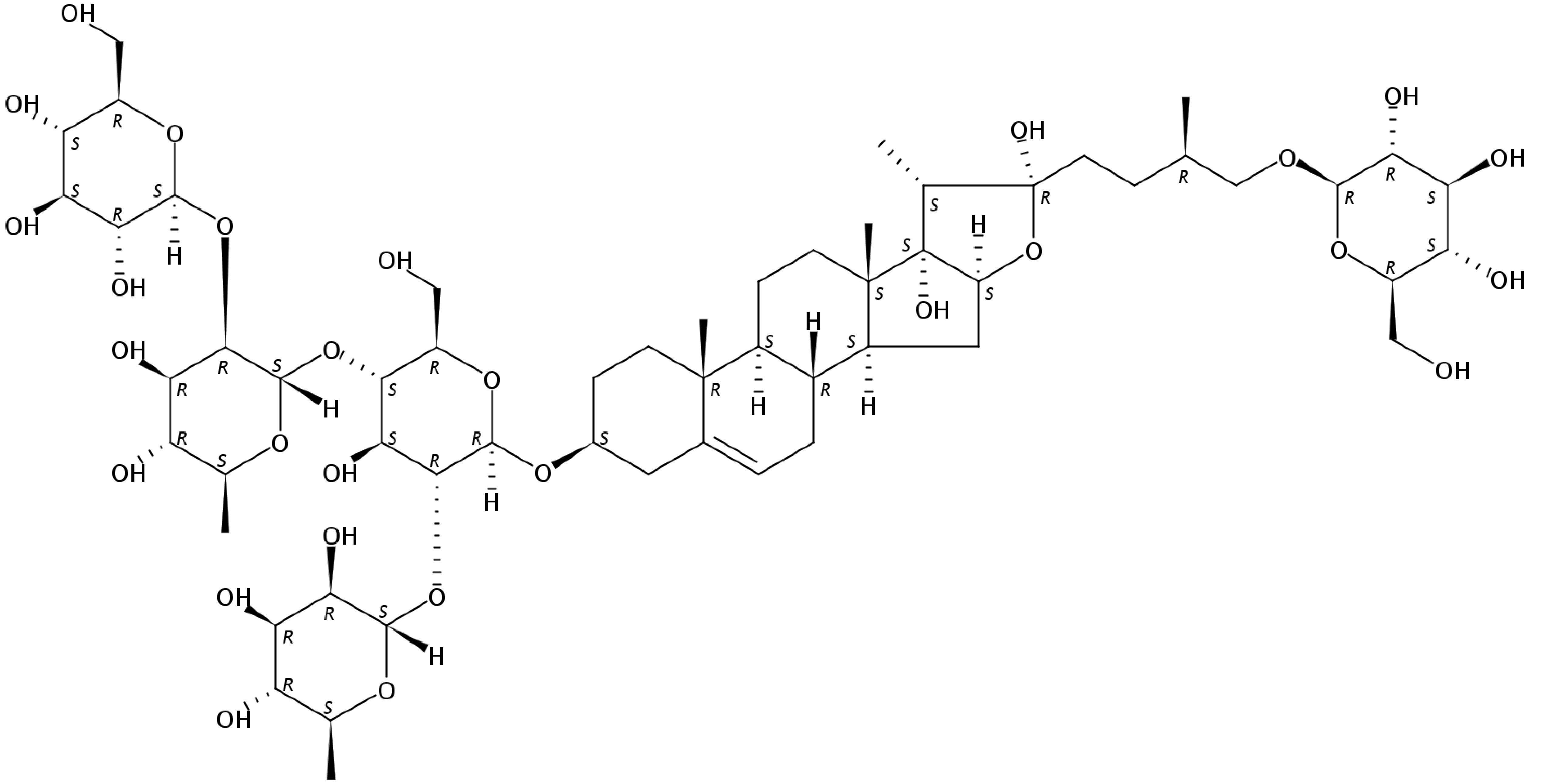

Hydroxysycophantine (***21***)

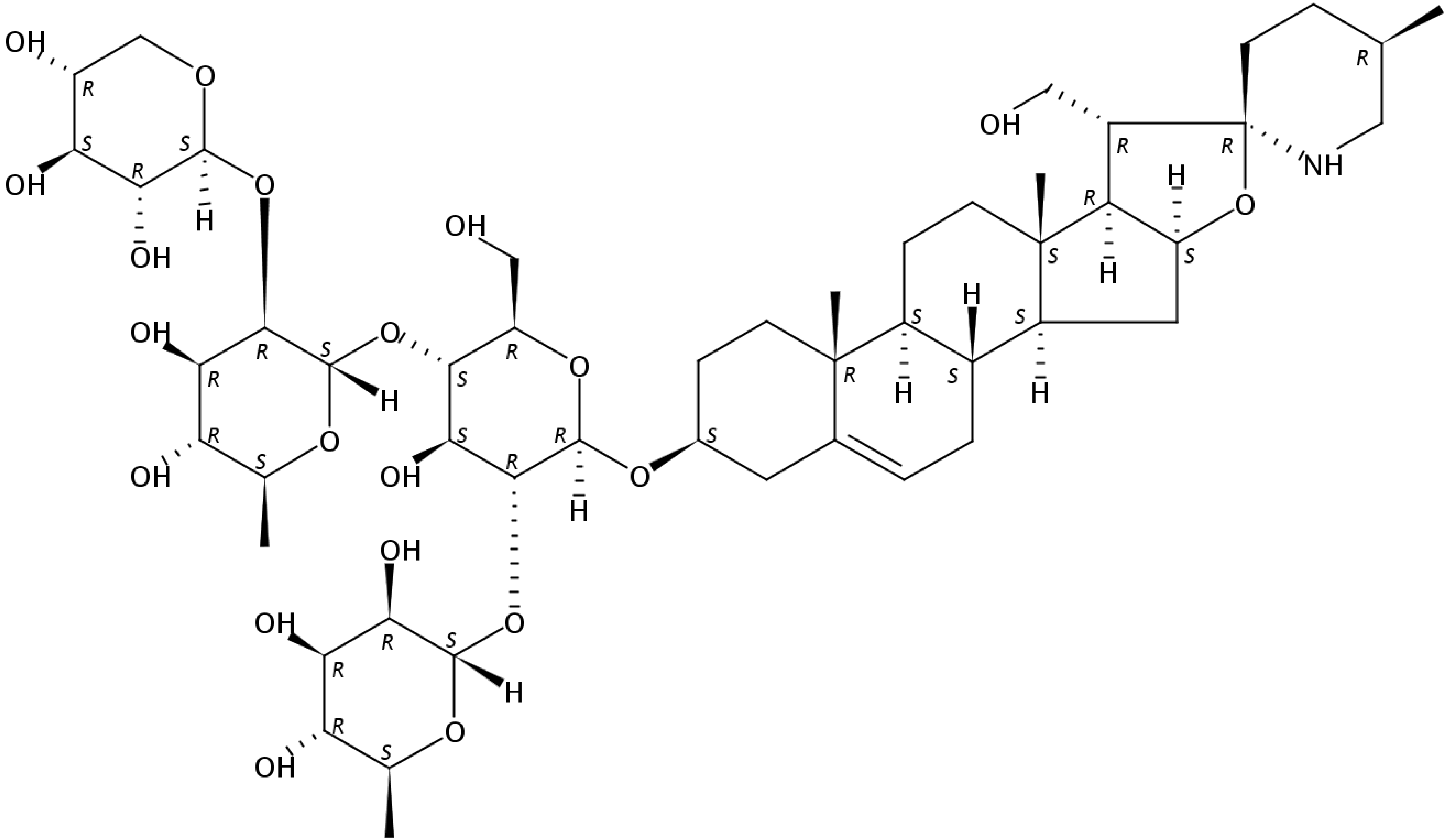

Malonyl-solamargine (***26***)

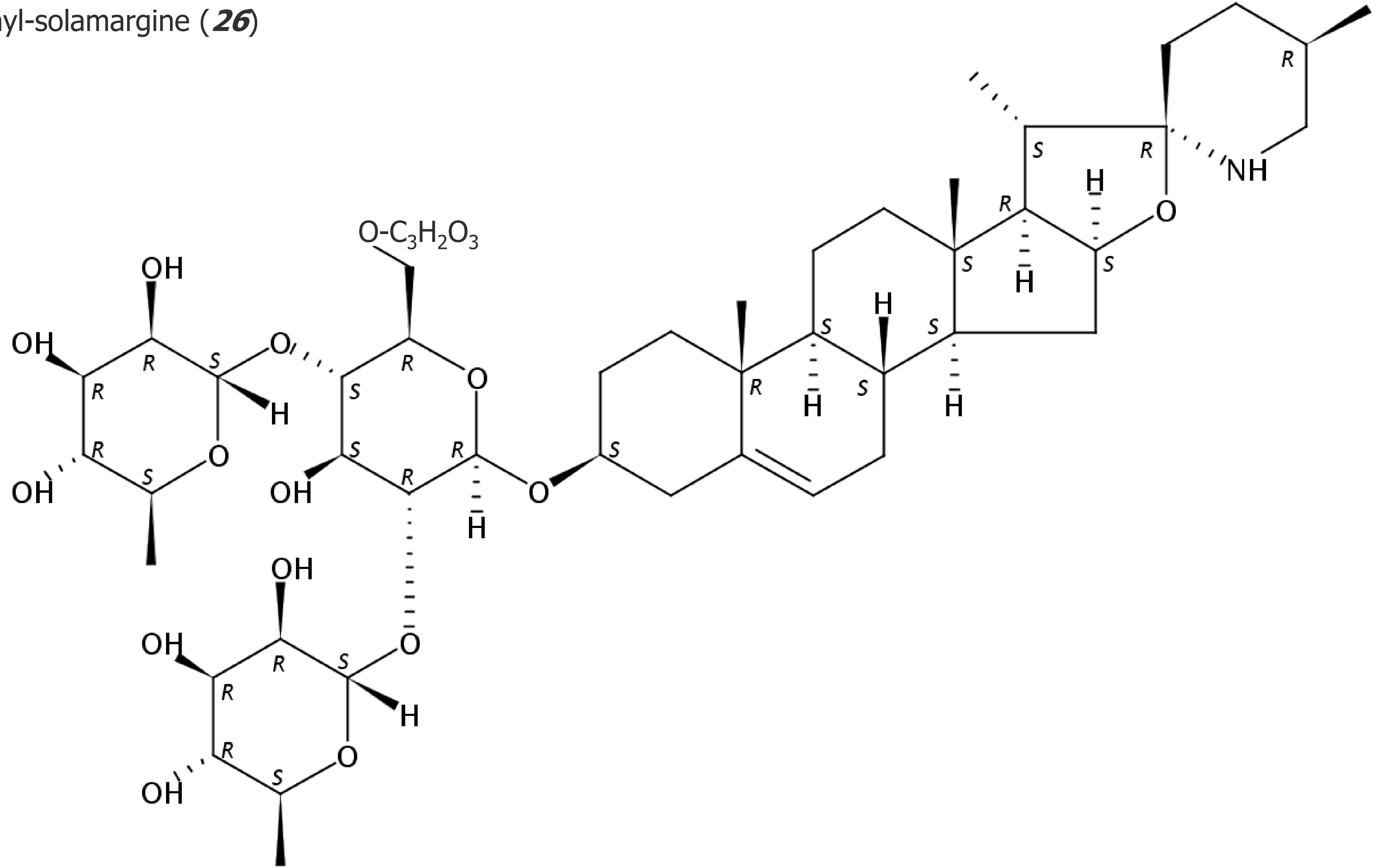

Solanigroside E (**28**)

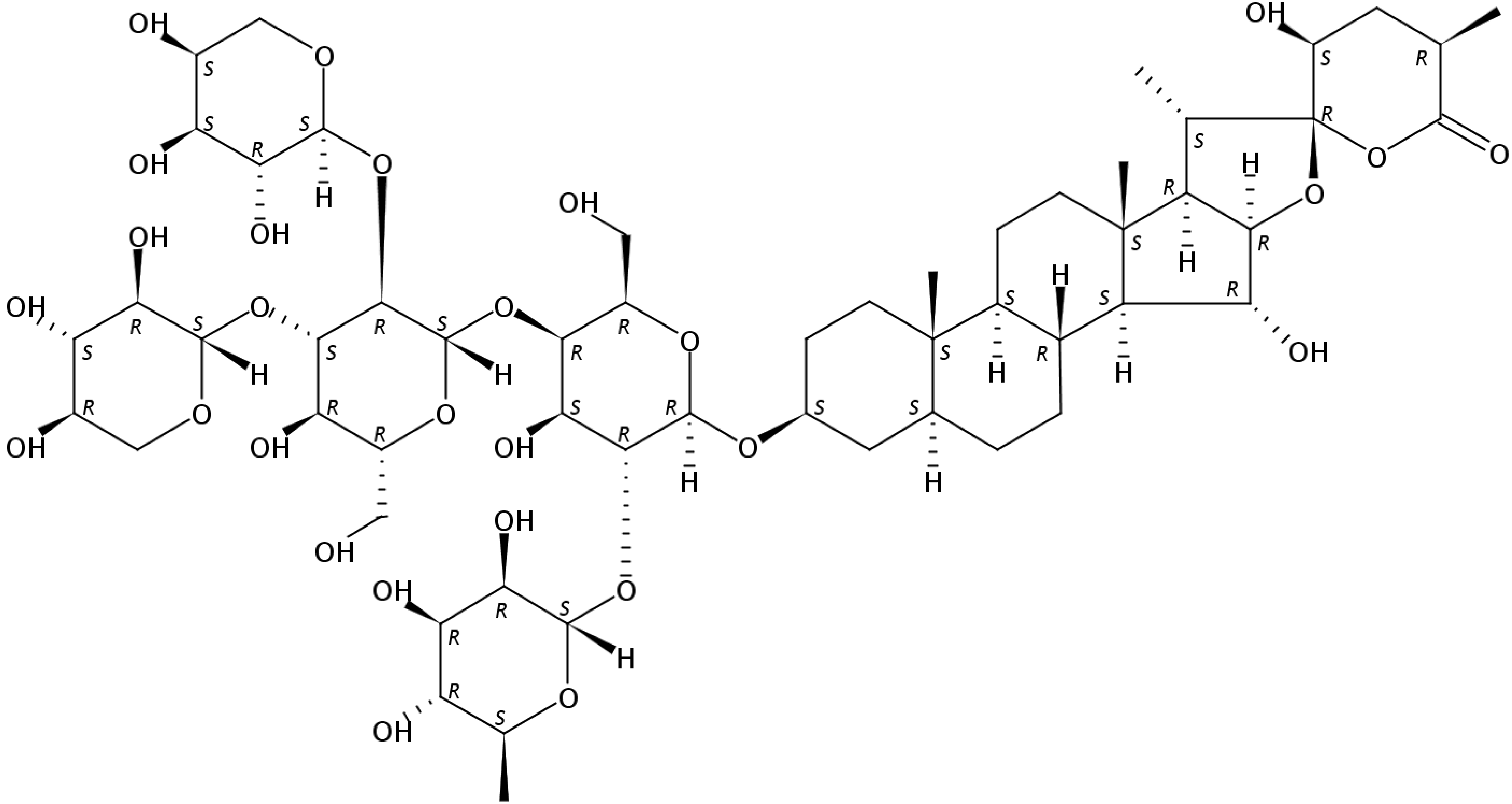

Anguivioside XI (***35***)

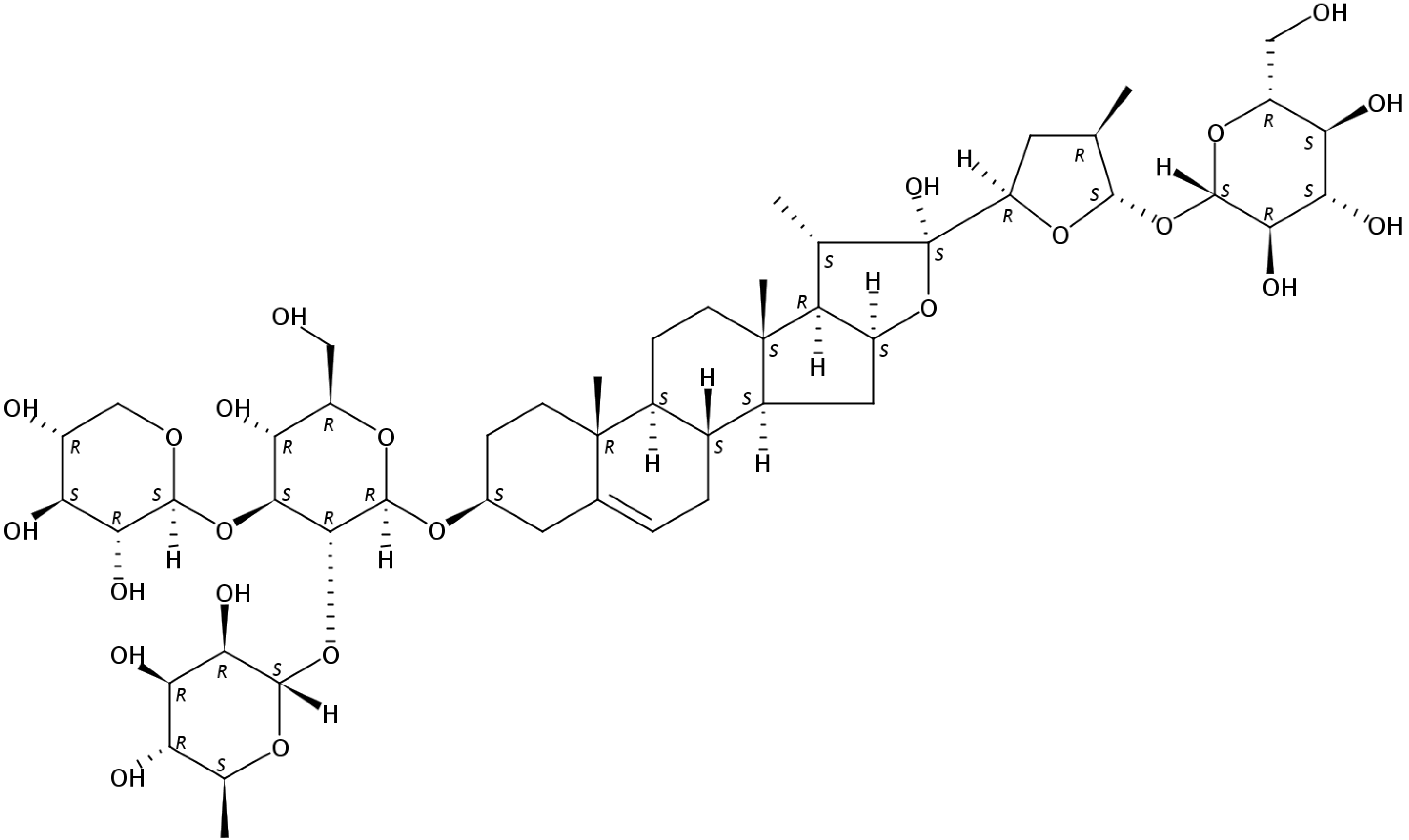

Lyconoside II (***40***)

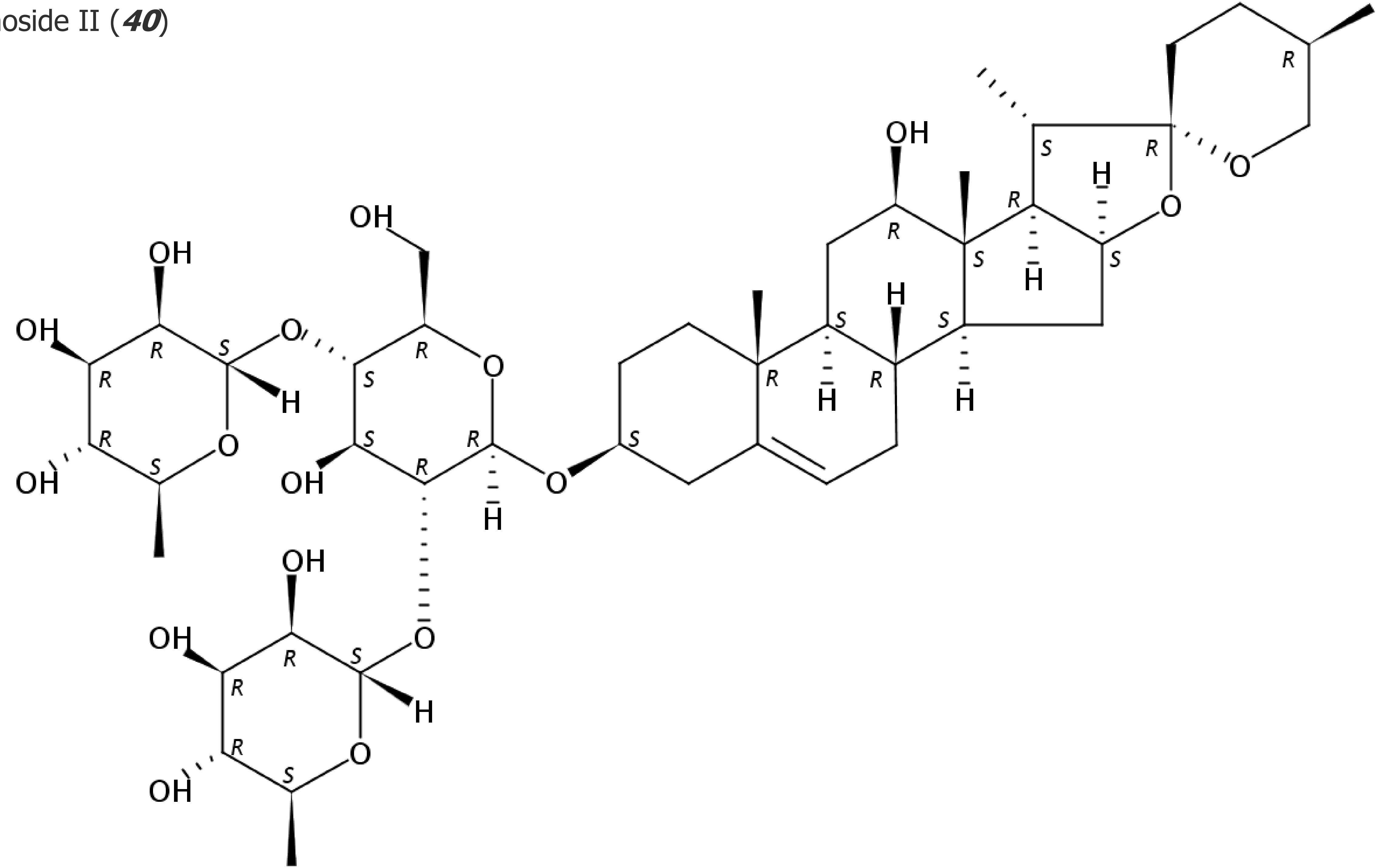

Solanigroside D (***43***)

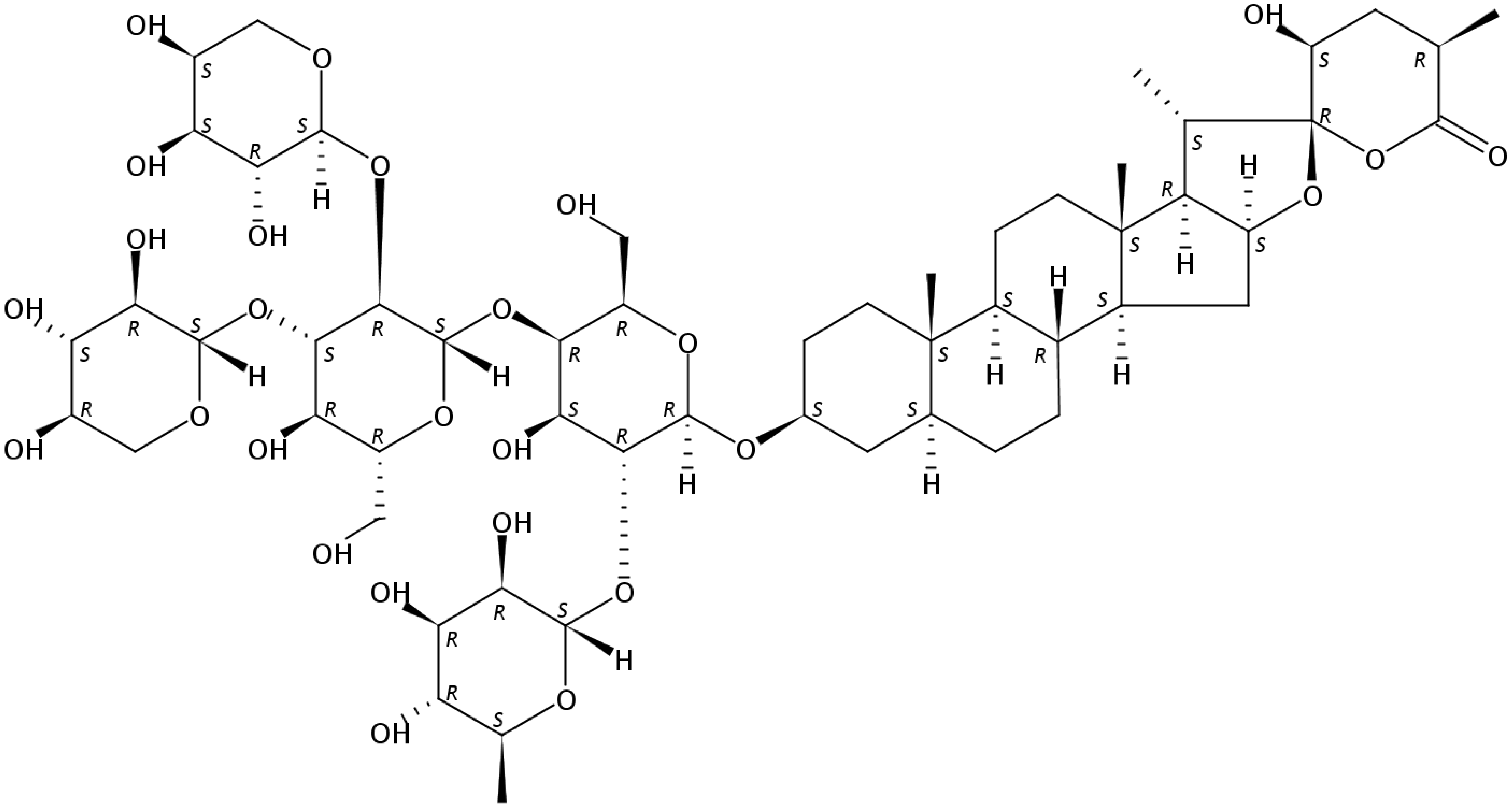

Supplement: Supplementary file 1 [file plants-11-00269-s001.zip › Figure S2.pdf]
