# Supplementary material for: High-Performance Liquid Chromatography–Mass Spectrometry Analysis of Glycoalkaloids from Underexploited Solanum Species and Their Acetylcholinesterase Inhibition Activity
Source: Plants (Basel). 2022 Jan 20;11(3):269. doi: 10.3390/plants11030269 (PMC8839269; doi:10.3390/plants11030269)
Supplement: Supplementary file 1 [file plants-11-00269-s001.zip › Table S1.pdf]

Tabel S1. Concentrations of individual glycoalkaloids detected in the selected *Solanum* species. Concentrations are expressed in ng g^{-1} on dry weight basis.

Glycoalkaloid:	**S. nigrum (OB)**			**S. nigrum (B)**			**S. melanocerasum**			**S. retroflexum**			**S. sisymbriifolium**			**S. quitoense**			**S. caripense**			**S. muricatum**		
	Roots	Steam	Leaves	Roots	Steam	Leaves	Roots	Steam	Leaves	Roots	Steam	Leaves	Roots	Steam	Leaves	Roots	Steam	Leaves	Roots	Steam	Leaves	Roots	Steam	Leaves
1	1921	91	53	36	4	1	11	5	5	221	59	31	489	13846	37549	79	734	225	1444	260	1512	31	17	86
2	764	39	25	2	1	1	1	0	1	80	20	10	33	272	30349	5	151	10	37	949	2373	1	16	113
3	1	0	1	1	1	1	1	1	1	1	4	3	1	8	51	11	1	1	67	94	17	2295	2320	195
4	0	1	1	1	1	1	1	1	1	2	0	1	4	1	2	380	0	1	39	3	5	18	36	17
5	44	17	7	47	34	12	17	8	5	14	7	6	3	15	33	173	1114	31	1378	12999	53247	268	20	1582
6	370	30	25	22	1	0	10	2	4	19	20	11	370	15690	2133	366	29	7	266	675	6890	26	25	175
7	23	4	8	20	2	1	51	28	11	90	10	9	1	1	1	41	8	1	175	29	46	66	17	1
8	53	266	20	204	562	81	35	14	5	30	11	58	41	4	125	113	18	1	13085	13008	84	2556	614	549
9	37	20	66	25	7	12	68	150	105	89	87	50	1	1	4	8	1	1	80	1	19	16	3	1
10	23	18	34	8	1	7	141	26	126	62	30	19	1	28	18	15	4	1	588	1	133	27	1	18
11	241	265	12	41	57	81	9	14	5	30	10	21	41	8	109	133	18	1	12656	24517	586	3206	2256	551
12	48	4	121	19	34	28	7	38	1	7	119	58	8	12	11	31	9	1	13822	4691	106	3865	377	131
13	15	24	120	19	34	28	7	38	1	8	115	3	9	15	11	37	9	1	13849	401	41	1236	252	40
14	164	34	12	33	52	10	8	5	2	15	1	9	22	77	50	64089	3403	552	12064	5244	198	593	662	82
15	272940	17416	9560	12858	123	61	758	70	54	19348	5562	3910	393	221	30	7049	6510	1975	11492	24066	85304	2342	2311	44554
16	44	808	64	583	1257	200	214	61	39	126	92	61	5808	29522	30713	8394	2654	202	165280	103414	28762	24937	40584	11536
17	11	56	61	28	12	23	34	76	9	35	37	47	1	1	76	11	1	1	3347	62	1	113	24	2
18	8	61	12	31	93	14	4	13	8	6	16	4	131	5383	5568	1419	452	37	23030	21014	5697	5329	1042	2197
19	4361	2241	36139	306	2113	7003	733	2039	285	1937	7475	4799	9	1	3	0	1	1	3	1	1	1	1	1
20	132316	22317	132675	28133	11752	10543	20850	11992	5821	25950	79084	53586	3	6	9	49	3	6	25	1	1	9	16	7
21	53	1	38	2	0	8	1	2	16	43	6	5	918	10683	4516	15149	4957	152	23317	12	34	34	12	15
22	363	628	1898	35	306	702	12	138	396	481	2054	1045	12	3	4	30	1	1	23	1	33	328	42	1
23	15	6	21	5	3	9	2	4	0	3	7	1	22	22	7	41491	3647	239	734	1	21	7	18	85
24	3733	1283	11569	158	820	4350	43	1170	7904	1425	5348	21346	1	1	6	209	28	1	27	11	14	33	1	11
25	255	39	954	33	149	103	68058	54865	6970	142	622	318	16	19	13	214	42	28	31	18	41	55	103	121
26	320583	18907	8214	1556	577	179	1793	103	70	24287	5136	3190	305	4899	139563	12289	20292	2309	14817	124445	562994	5591	11078	182798
27	943	1208	14576	1577	643	2810	1077	1025	1908	8525	5658	3840	1	1	21	36	1	1	88	3	9	370	38	2
28	111252	87378	364962	6034	27179	57636	4403	31179	54252	52101	163719	287474	5	9	41	77	14	21	22	5	14	23	12	17
29	2669	1612	2057	861	456	177	22	325	87	427	1835	1156	131	10658	8092	2277	4730	106	80	82	63	1	12	6
30	9578	484	3110	598	48	78	616	321	27	2899	225	321	1	1	1	1	1	1	1	1	1	1	1	1
31	165	115	173	42	42	8	225	569	25	141	130	73	259	25	8	24218	2001	133	9596	516	52	131	57	55
32	1572	70	132	24	139	59	472	2957	65	1264	523	281	2289	13025	13911	27916	181	68	2535	3220	6092	2135	33	173
33	81	63	214	43	17	24	184	210	21	64	84	35	13	16	17	32	1	1	30	13	31	35	9	2
34	2645	1001	1818	683	252	98	1	282	69	5085	1216	891	1	8	18	27	1	1	8	23	44	500	0	2
35	1010	536	3527	325	150	46	1633	2280	31	602	800	279	49	9	13	103	162	12	1976	113	8	75	31	16
36	447	36	265	218	207	148	100	2275	86	899	1142	658	393	2341	2397	5241	36	15	552	519	1082	393	8	35
37	49	41	181	24	16	16	118	145	12	39	69	54	7	7	11	1	62	1	29	7	1197	300	47	2
38	4109	444	5023	1590	86	103	4024	608	5	9243	35	22	1	1	1	7	1	1	1	1	1	1	4	1
39	309384	18519	8132	1573	38	179	1805	32	69	23847	5104	3172	304	4760	170	12182	19771	2286	4903	119755	2485	323	4307	1206
40	238	60	182	156	23	8	315	282	14	857	59	21	195	644	379	837	2256	62	2909	124	414	328	83	6
41	919	28	11	29	23	2	19	2	1	231	169	8	35	85	3173	10894	83	57	1	108	669	25	21	1218
42	1159	39	54	20	69	35	8	2940	29	622	40	21	18	321	20410	17	4	1	1	54	113	30	3	6
43	1284	448	3247	515	174	56	375	370	34	1295	547	90	1	6	13	25	1	1	1	1	5	1	1	1
44	20	3	17	17	131	35	50	2	1	60	452	6	1	1	1	0	1	1	1	1	1	1	1	1
45	20	69	407	44	46	3	164	390	10	189	44	248	109	1	235	33819	29	63	8	194	681	30	9	4
46	122994	34439	113094	14012	2048	2320	17724	6436	220	79095	11602	13597	1	8	3	71	2	4	1	1	1	13	25	5
47	139219	31829	121888	11907	1566	1400	15173	6436	220	67530	8628	10214	2	8	3	71	2	4	1	1	1	13	25	5
48	4	1	2	3	0	1	1	1	1	16	5	7	24	1	118	19866	52	62	1	29	29	34	85	9
49	3	4	60	47	21	12	11	1	1	163	23	6	1865	31	1	11	1	1	18	7	1	8	1	1
50	5	5	36	0	9	17	1	0	1	2	10	9	4020	19	18	5	1	1	76	11	75	2	1	1
51	161	34270	98267	61	243	621	1	9	1	378	3297	3913	1	1	1	1	1	1	10	1	1	1	1	1
